# Spike Detection for Large Neural Populations Using High Density Multielectrode Arrays

**DOI:** 10.3389/fninf.2015.00028

**Published:** 2015-12-18

**Authors:** Jens-Oliver Muthmann, Hayder Amin, Evelyne Sernagor, Alessandro Maccione, Dagmara Panas, Luca Berdondini, Upinder S. Bhalla, Matthias H. Hennig

**Affiliations:** ^1^Manipal UniversityManipal, India; ^2^Department of Neurobiology, National Centre for Biological Sciences, Tata Institute of Fundamental ResearchBangalore, India; ^3^School of Informatics, Institute for Adaptive and Neural Computation, University of EdinburghEdinburgh, UK; ^4^Department of Neuroscience and Brain Technologies, Istituto Italiano di TecnologiaGenova, Italy; ^5^Institute of Neuroscience, Newcastle UniversityNewcastle, UK

**Keywords:** spike detection, microelectrode array, cultured neurons, retina, correlations

## Abstract

An emerging generation of high-density microelectrode arrays (MEAs) is now capable of recording spiking activity simultaneously from thousands of neurons with closely spaced electrodes. Reliable spike detection and analysis in such recordings is challenging due to the large amount of raw data and the dense sampling of spikes with closely spaced electrodes. Here, we present a highly efficient, online capable spike detection algorithm, and an offline method with improved detection rates, which enables estimation of spatial event locations at a resolution higher than that provided by the array by combining information from multiple electrodes. Data acquired with a 4096 channel MEA from neuronal cultures and the neonatal retina, as well as synthetic data, was used to test and validate these methods. We demonstrate that these algorithms outperform conventional methods due to a better noise estimate and an improved signal-to-noise ratio (SNR) through combining information from multiple electrodes. Finally, we present a new approach for analyzing population activity based on the characterization of the spatio-temporal event profile, which does not require the isolation of single units. Overall, we show how the improved spatial resolution provided by high density, large scale MEAs can be reliably exploited to characterize activity from large neural populations and brain circuits.

## 1. Introduction

Emerging generations of high-density microelectrode arrays (MEAs) based on CMOS technology allow recording extracellular signals from neural population activity with unprecedented detail (Eversmann et al., 2003; Berdondini et al., 2005; Hutzler et al., 2006; Maccione et al., 2014). These devices are capable of simultaneously recording extracellular activity with thousands of channels at near cellular resolution, providing an unbiased sample of neural activity in a variety of *in vitro* preparations. Such recordings have the potential to deliver unique new insights into network dynamics, but their analysis is challenging both due to signal properties that differ substantially from conventional arrays and the vast amounts of raw data they generate.

The intrinsic noise level of the BioCam platform (3Brain GmbH) used here is about 11 µV_*rms*_ for single channel readouts (Imfeld et al., 2008), comparable to values reported for high density arrays with few electrodes (Prentice et al., 2011; Marre et al., 2012). For whole chip recordings, it increases to about 26 µV_*rms*_ due to aliasing of high frequencies when multiplexing the signals. Much lower noise levels of 2.4 µV_*rms*_ were reported for MEAs where a high electrode density was combined with a relatively lower number of amplifiers (Frey et al., 2010; Müller et al., 2015). These arrays are now capable of recording simultaneously from up to 1024 electrodes. The same noise level is achieved by conventional arrays, but these use larger electrodes and therefore average signals over a larger volume. The small electrode dimensions in high density arrays enable the detection of smaller current sources (e.g., from axons) if the resistivity of the preparation is high. Conversely, even though distant current sources will be picked up by many electrodes, their detection is impaired because of a higher intrinsic noise level.

In addition, recording 4096 channels sampled at 8 kHz with 12 bit resolution yields data rates of about 0.37 GBit/s, or 2.8 GB/min. These high data rates become particularly challenging for prolonged continuous recordings over several hours or days. Neural activity, on the other hand, is usually rather sparse. Firing rates in cultured networks or other isolated preparations rarely exceed 10 Hz, and rate distributions are typically skewed toward values well below 1 Hz (Griffith and Horn, 1966; Hromádka et al., 2008). On high density arrays with an electrode size of 21 × 21 μm^2^, each channel records the activity of only few neurons due to spatial constraints. Hence the rate of relevant signals is at most 10 MB/min and typically lower since often not all channels record neural activity.

Therefore, a spike detection algorithm needs to be highly efficient in order to process large datasets in a short time and extract the most relevant information for a consecutive spike sorting. It has to be designed to detect spikes in a narrow volume close to the electrodes and ignore a high amount of background activity and noise. The algorithm must automatically estimate the voltage baseline, since it may vary across electrodes and in time. Additionally, the fabrication process does not guarantee an equal performance for all electrodes such that robustness to electrode failures is required.

For conventional arrays, raw voltage traces from extracellular recordings are typically high-pass filtered with a cutoff frequency between 300 and 800 Hz (Quiroga et al., 2004; Jäckel et al., 2012; Kadir et al., 2014; Swindale and Spacek, 2014). For high electrode densities, and when spike shapes are known, algorithms can reduce or avoid high-pass filtering at some point in the analysis (Nenadic and Burdick, 2005; Prentice et al., 2011; Marre et al., 2012). For detecting and sorting spikes, one way is to identify candidate events which are then used to create templates and a subsequent fitting of such templates to the raw voltage traces. Template matching can be implemented in a highly efficient way on FPGAs (Dragas et al., 2015), but relies on an assumption that spikes from the same neuron will only change in amplitude and the generation of templates often requires a considerable amount of manual intervention. For efficient detection of data from high density arrays without using templates, Gibson et al. (2010) proposed using the Teager Energy Operator for sampling rates of 24 kHz and when hardware dependent noise sources can be neglected, and found a good performance for a threshold based approach as well. Swindale and Spacek (2014) used a colored Gaussian noise matched to recordings and found that thresholding a peak-to-peak difference works best for high-pass filtered data (cutoff at 500 Hz).

Threshold-based detection can be supplemented by defining constraints on the spike shape, such as a repolarization following the main signal or non-linear filters, which require some previous knowledge of the expected spike shape (Kim and Kim, 2000). It can be highly efficient when combined with continuous noise estimation. It can be realized in real-time during acquisition even on large arrays (Maccione et al., 2009) and implemented in hardware, for instance using wavelet based compression and feature extraction (Imfeld et al., 2009). Once detected, putative spikes are then clustered according to spike shape parameters to separate multiple neurons recorded by the same channel and to exclude false positives (Lewicki, 1998; Einevoll et al., 2012).

In high density recordings signals from the same neuron are mostly detectable on multiple channels. While this redundancy complicates assigning individual waveforms to different neurons, it can be exploited to improve the signal-to-noise ratio (SNR) and detection (Franke et al., 2012), as also done for tetrode or polytrode recordings (Gray et al., 1995; Harris et al., 2000; Mechler et al., 2011; Marre et al., 2012; Rossant et al., 2015). A clustering and a spatio-temporal lockout method to remove duplicates in the detection was compared by Swindale and Spacek (2014) with similar performance for both methods. In their paper, they only found spikes on up to 10 electrodes, but for a higher electrode density, multiple detections would be common, and a requirement on the number of detections could be a way to exploit the spatial spread of signals.

Here we present a set of methods for efficient and reliable event detection and classification of spikes specifically designed for use on large scale, high density recordings. We used data recorded with the 4096 channel Active Pixel Sensor (APS) MEA (Berdondini et al., 2005, 2009), with an electrode center to center spacing *d*_*pitch*_ of 42 µm. Instead of high-pass filtering, we employed online estimates of percentiles of the voltage traces to estimate the baseline and noise level. Since the strongest current during a spike should originate from the axon hillock of the neuron, we expected that spike current sources should be localizable. Therefore, we used a family of spatial (but not temporal) templates covering different source locations to approximate the spatial profiles of spikes, instead of estimating templates directly from amplitudes in the raw data. Assessment of the performance was performed independently using synthetic data, by imaging of an antibody labeled preparation, and by exploiting correlations in neural activity. We expect that these methods are applicable without much modification to data from similar systems. All code to replicate the analysis shown in this paper is provided at https://github.com/martinosorb/herding-spikes, and example datasets at https://portal.carmen.org.uk/#link=URN:LSID:portal.carmen.org.uk:metadata:42944.

## 2. Materials and methods

### 2.1. Neurophysiology

High density recordings from the neonatal mouse retina and cultured dissociated hippocampal neurons were performed using the BioCam4096 platform with APS MEA chips type BioChip 4096S (3Brain GmbH, Switzerland), providing 4096 square microelectrodes (21 × 21 µm) on an active area of 2.67 × 2.67 mm, aligned in a square grid with 42 µm spacing. The platform records at a sampling rate *f*_*s*_ between 7–8 kHz/electrode when measuring from the full 64 × 64 channel array (7563 Hz retina, 7702 Hz culture). Raw data were visualized and recorded with the BrainWave software provided with the BioCam4096 platform. Activity was recorded at 12 bits resolution per channel, low-pass filtered at 5 kHz with the on-chip filter and high-pass filtered by setting the digital high-pass filter of the platform at 0.1 Hz.

Experiments were performed on neonatal (P2-15) C57bl/6 (http://jaxmice.jax.org/strain/000664.html) mice in Newcastle, conducted and approved under the UK Home Office, Animals (Scientific procedures) Act 1986, and on cultured neurons in Genova, where all procedures involving experimental animals were approved by the institutional IIT Ethic Committee and by the Italian Ministry of Health and Animal Care (Authorization ID 227, Prot. 4127 March 25, 2008).

#### 2.1.1. Cultured neurons

Neural cultures were obtained by dissociating hippocampal neurons from embryonic rat brain tissue at day E 18 and plating the cells on APS MEAs pretreated with adhesion factors (PEI). Subsequently, cultures were incubated in Neurobasal supplement with 1% Glutamax 2% B-27 medium, in a humidified atmosphere 5% CO_*2*_ at 37°C. Fifty percent of the medium was changed every 6 days. The data presented in this study were 20 min long recordings from two cultures aged 21 days *in vitro* (DIV) and 18 DIV (for the imaged culture).

For the analysis of spatial patterns, 15 min long recordings from three cultures aged 21 DIV at the beginning of the experiment were used. A low dose (5 µM) of CNQX (6-cyano-7-nitroquinoxaline-2,3-dione) was applied to two of these cultures after an initial baseline recording and washed out by twice replacing 2/3 of the culture media after 48 h.

The staining of low density cultures was performed as described by Ullo et al. (2014). After recording, cells were fixed at room temperature in 4% paraformaldehyde, permealized and incubated for 2 h with primary antibodies anti-b3 tubulin (Sigmaaldrich) and anti-NeuN (Chemicon Millipore). Successively, secondary antibodies Alexa-Fluor488 and Alexa-Fluor 546 conjugated were added to label, respectively b3-tubulin and NeuN. Fluorescent images at 20x magnification were acquired by means of an upright microscope Olympus BX51 and stitched together. The result is a high resolution image of the entire culture recorded by the 2.7 by 2.7 mm^2^ active area of the chip.

#### 2.1.2. Neonatal retina

Mouse pups were killed by cervical dislocation and enucleated prior to retinal isolation. The isolated retina was placed, retinal ganglion cell (RGC) layer facing down, onto the MEA. Coupling between the tissue and the electrodes was achieved by placing a small piece of polyester membrane filter (Sterlitech, Kent, WA, USA) on the retina followed by a custom made anchor. The retina was kept at 32°C with an in-line heather (Warner Instruments) and continuously perfused using a peristaltic pump (~1 ml/min) with artificial cerebrospinal fluid (aCSF) containing the following (in mM): 118 NaCl, 25 NaHCO_*3*_, 1 NaH_*2*_ PO_*4*_, 3 KCl, 1 MgCl_*2*_, 2 CaCl_*2*_, and 10 glucose, equilibrated with 95% O_*2*_ and 5% CO_*2*_. Retinas were allowed to settle for 1–2 h before we started the recordings to allow spontaneous activity to reach steady state levels. The tissue was constantly perfused with fresh aCSF. The recording shown in this paper was 30 min long and from a retina at postnatal day 11. A further recording of 15 min duration was done from the same chip after removing the retina (empty chip recording).

### 2.2. Event detection (online method)

**Figure 1:**
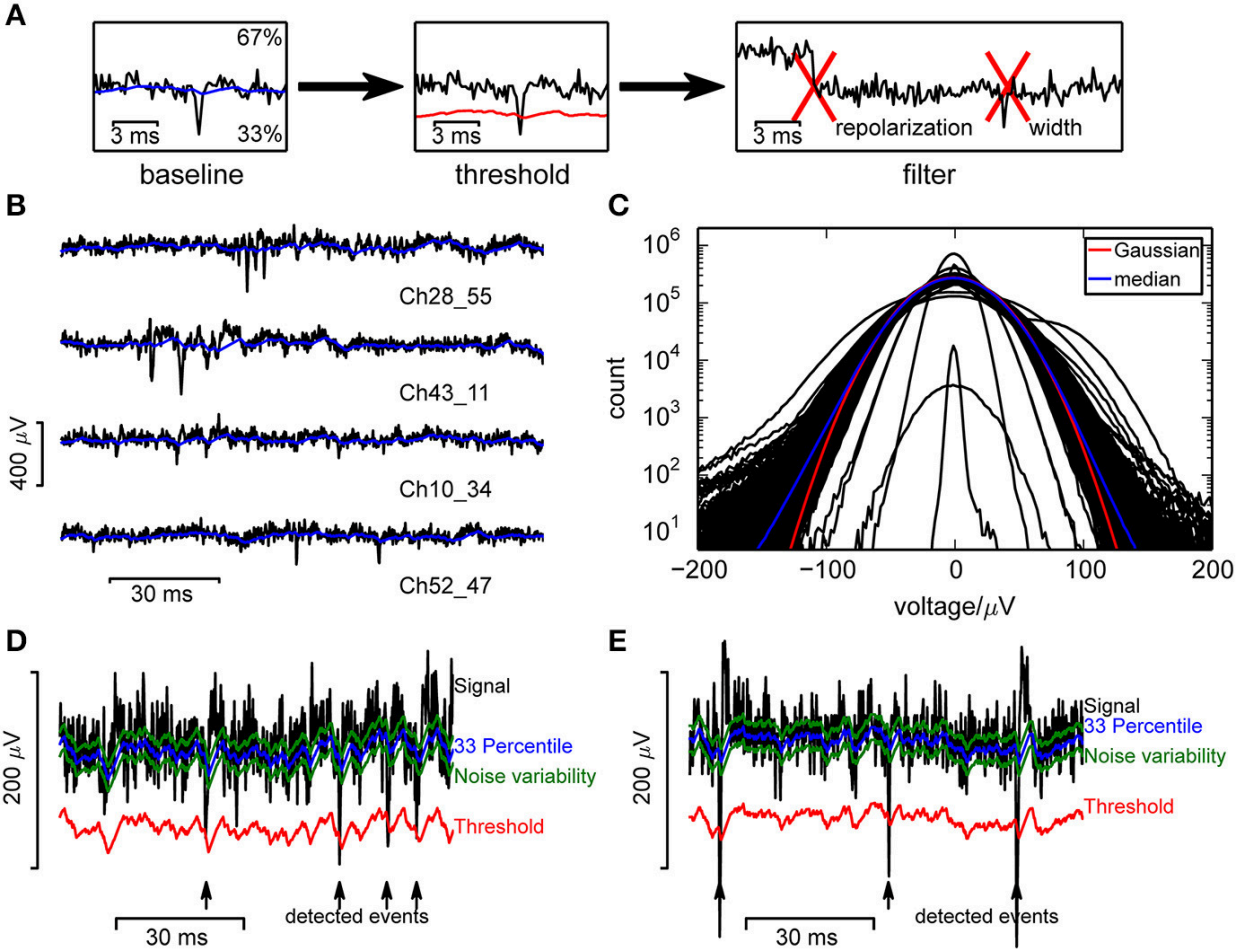
**Event detection in single channels**. **(A)** Illustration of the algorithm. **(B)** Raw voltage traces from a selection of channels, from a recording from a culture. **(C)** Voltage histograms from a random selection of 400 channels from a single recording show that recorded signals followed a broad distribution and could be highly variable between channels. A Gaussian distribution (red curve) was fitted to the median (blue) across all channels. **(D,E)** Measures used in the online spike detection method, illustrated for a recording from a culture **(D)** and retina **(E)**.

Spikes detection relied on a variable signal threshold, which was computed as follows. First, to compensate for correlations of the median voltage *x*_*global*_ across all channels, a likely consequence of external fields, the median voltage *x*_*global*_ was subtracted from the raw signals *x*. Then the algorithm used two variables which represented an online estimate of a baseline *b* (the local 33rd percentile) and a variability estimate *v* around the baseline. They were both updated incrementally as follows (Figures 1D,E):

- *b* was increased by 1/2 *f*_*b*_∕*f*_*s*_ (cf. Table 1) if the raw signal exceeded *b* + *v* and decreased by *f*_*b*_∕*f*_*s*_ if the raw signal was below *b* − *v*. This adjustment was not symmetrical in order to ignore irrelevant positive fluctuations.
- *v* was decreased by *f*_*v*_∕*f*_*s*_ (cf. Table 1) if the signal was within the interval (*b* − *v, b*] or, to compensate for spikes, (−∞, *b* − 6*v*], and increased by the same amount for signal amplitudes (*b* − 5*v, b* − *v*].

**Table 1:** **Detection parameters used in the examples illustrated in this paper**.

**Parameter**	**Value**	**Description**
**ONLINE SPIKE DETECTION**		
*f*_*s*_	7–8 kHz	Sampling rate
*d*_*pitch*_	42 µm	Electrode pitch
*f*_*v*_	0.03125 *f*_*s*_ μ*V*	Variability update rate
*f*_*b*_	0.5 *f*_*s*_*v*	Baseline update rate
θ	6	Detection threshold
θ_*ev*_	10.5	Minimum depolarization area
τ_ev_	0.27 ms (2 frames)	Interval for depolarization area
τ_event_	1ms (7 frames)	Maximum depolarization width
θ_*b*_	0	Repolarization threshold
**SPATIAL INTERPOLATION, ADJUSTABLE PARAMETERS**		
θ	5	Detection threshold
θ_*b*_	0	Repolarization threshold
**SPATIAL INTERPOLATION**		
τ_event_	1 ms (7 frames)	Characteristic event length
τ_coinc_	±0.27 ms (2 frames)	Window for coincident events
*w*_cs_	4/1	Center/surround weighting
τ_pre_	1 ms (7 frames)	Cut-out window before peak
τ_post_	2.2 ms (15 frames)	Cut-out window after peak

The threshold for event detection was then set to *b* − θ*v*, deflections in the positive direction were ignored. The 33rd percentile was a better baseline estimate than the signal median, as it was less affected by such events.

After threshold crossing, three shape criteria were applied to reduce the number of false positives. Events were accepted when:

- the sum of all baseline subtracted voltages from the threshold crossing up to τ_ev_ after the peak was larger in amplitude than (−θ_ev_*v*).
- there was no larger minimum for the subsequent τ_event_ interval after the peak.
- at least one frame in a τ_event_ interval after the peak was larger than *b* + θ_*b*_*v*, indicating presence of a repolarization.

This initial detection could be performed online during an ongoing recording. For the subsequent analysis, channel ids, spike time stamps and spike amplitude (in units of the current variability estimate *v*) were stored. The parameters used for the analysis shown in this paper are given in Table 1.

### 2.3. Event detection (spatial interpolation)

In our system, spikes often produced a high amplitude only for less than 0.5 ms (2–3 frames at 7 kHz) and on 1–4 electrodes. If the true spatio-temporal profile of a spike was known, a dot product of the raw voltage traces and such templates could be employed to efficiently detect spikes (Marre et al., 2012; Dragas et al., 2015). However, estimating such templates required a high firing rate, large amplitudes, or long recordings (and would be biased toward detecting units satisfying those criteria) and was computationally demanding. In order to avoid the prior estimation of templates, but to still account for the spatio-temporal spread of the signal, we added a set of virtual channels centered between the real electrodes. Spatial averages of the signals on the true channels were then computed with two different weightings, to account both for event sources close to and between recording channels.

*Five-channel interpolation*. The current source was assumed to be close to an electrode. We computed a weighted signal of a group of five nearest neighbor channels, where the center channel contributed *w*_cs_∕(3 + *w*_cs_) of the amplitude, and the three largest amplitudes of the four surrounding channels were each weighted by 1∕(3 + *w*_cs_). This was done for each frame and the variability estimate was weighted in the same manner. This signal then replaced the original signal in each channel.

*Four-channel interpolation*. The current source was assumed to be roughly centered in a 2 × 2 grid of four channels. The three largest absolute signal amplitudes and corresponding variability estimates were averaged. This value was then added as a virtual channel between true channels.

The specific weights do depend on the inter-electrode distance *d*_*pitch*_ and are therefore system specific. To facilitate adaptation of the method for other systems we studied that dependence in a simplified setting in Supplementary Figure 1. Discarding the lowest amplitude in both scenarios was done for two reasons. First, it was a simple way to effectively implement four different templates for each scenario and ensuring that any event was only detected once. As a result, the location of the current source would have a lower impact on the detection performance. Second, as usually not all electrodes performed equally well, this procedure could reduce the influence of positive outliers if they occurred on single electrodes and were clearly unphysiological. Of course, this approach would have a higher occurrence of false positives as it relied on multiple testing, but we argued that events detected from the two types of interpolated signal could subsequently be distinguished by their spatial position.

For the temporal processing, we essentially followed the same procedure as for the online algorithm, but averaged signals over 0.4 ms and also subtracted a running estimate of signal fluctuations that could be explained by the voltage median *h*_*i*_ · (*x*_*global*_(*t*) − *x*_*global*_(*t* − 1)). The hidden variable *h*_*i*_ was incremented with a speed of 1/s when the corrected voltage on individual electrodes *x*_*i*_(*t*) − *x*_*global*_(*t*) − *h*_*i*_ · (*x*_*global*_(*t*) − *x*_*global*_(*t* − 1)) changed in the same direction as global voltage fluctuations *x*_*global*_ and decremented otherwise. This method differed from the approach in Marre et al. (2012) where a covariance matrix between all channels was determined to whiten the signals, which assumed temporal stationarity over the whole recording.

Baseline subtracted signals were then normalized by the variability estimate for each channel, and the minimum over two consecutive frames was taken as event peak to account for a potential temporal shift between neighboring channels. When this signal crossed a threshold −θ, a local minimum was accepted as a spike when there was no larger minimum for the subsequent τ_event_ interval, and no larger local minimum was found in one of the eight neighboring locations (four virtual and four real channels) in an interval of length τ_coinc_ around the peak. If at least one frame in the τ_event_ interval had a value above θ_*b*_ (i.e., baseline), the event was marked as a repolarizing event.

#### 2.3.1. Spatial origins of events

We assumed that, at least at short distances, the potential falls off roughly as ~ 1∕*r*^2^ to ~ 1∕*r* with distance *r*, so that the amplitudes at neighboring electrodes could be used to estimate the current source locations (Pettersen and Einevoll, 2008; Lindén et al., 2011; Prentice et al., 2011). To this end, raw data cut-outs from a set *I*_*cutout*_ of 9–12 channels with spatial coordinates ξ_*i, x*_ and ξ_*i, y*_ (*i* ∈ *I*_*cutout*_) were baseline subtracted and the peak value was taken as amplitude for each channel.

A naive estimate of the barycenter of these amplitudes was biased toward the center of the cut-out region whenever the amplitude estimate was offset. For our system, this problem could not be resolved by increasing the size of the cut-out region and estimating that offset, as distant electrodes would have a low SNR, but a strong influence on a barycenter estimate. We therefore compromised on the location bias to reduce the spatial spread of location estimates from the same source. To this end, the median amplitude was subtracted, the resulting amplitudes clipped at zero, and the center of mass computed as an estimated current source location.

The detailed procedure to estimate the current source location for a single event from raw signals in multiple, neighboring channels is outlined in Algorithm 1, and had the following steps:

**Algorithm 1:** Localization of events.

1:	**for** *i* in *I*_cutout_ **do**
2:	${\tilde{x}}_{i}\left(t\right)=\left(x_{i}\left(t\right)-x_{global}\left(t\right)-\langle h_{i}\rangle \cdot \left(x_{global}\left(t\right)-x_{global}\left(t-1\right)\right)\right)∕\langle v_{i}\rangle$
3:	*z*(0 : τ_pre_/2) = *b_i_*
4:	*z*(τ_pre_/2 : τ_pre_) = *x*(*t*_0_ − τ_pre_ : *t*_0_ − τ_pre_/2)
5:	*z*(τ_pre_ : τ_post_) = *x*(*t*_0_ + τ_pre_ : *t*_0_ + τ_post_)
6:	$A_{i}={\text{max}}_{t}\left(\left(\text{median}\left(z\right)-{\tilde{x}}_{i}\left(t\right)\right)*\left[\frac{1}{6},\frac{1}{3},\frac{1}{3},\frac{1}{6}\right]\right)$
7:	**end for**
8:	$A_{m}\text{ = median(}A_{I_{\text{cutout}}}\text{)}$
9:	$B_{j}=\underset{i}{\sum }\left(\text{clip}\left(A_{i}-A_{m},0,\infty \right)\cdot \xi_{i,j}\right)∕\underset{i}{\sum }\left(\text{clip}\left(A_{i}-A_{m},0,\infty \right)\right),\;i\in I_{cutout},\;j\in \left\{x,y\right\}$
10:	**for** *i* in *I*_cutout_ **do**
11:	$A_{i}=A_{i}\cdot \text{clip}\left(2-\sqrt{{\left(B_{x}-\xi_{i,x}\right)}^{2}+{\left(B_{y}-\xi_{i,y}\right)}^{2}},0,1\right)$
12:	**end for**
13:	${\hat{B}}_{j}=\underset{i}{\sum }\left(A_{i}\cdot \xi_{i,j}\right)∕\underset{i}{\sum }\left(A_{i}\right)$
14:	$Â=\underset{i}{\sum }\left(A_{i}\right)∕2$

*Baseline*. First the global fluctuations and the average fraction of global fluctuation increments explaining the signal were subtracted to reduce global correlated fluctuations, which would interfere with the localization. In order to put less weight on noisy channels, signals were divided by the variability *v*_*i*_ (*i* ∈ *I*_*cutout*_) temporally averaged over the whole recording.

Next, we subtracted the baseline voltage of individual channels as the median of an array *z* which contained (⌊_pre_ *f*_*s*_)∕2⌋) frames with the online baseline estimate at the time of the detection of the spike, and the initial (⌊(τ_pre_ *f*_*s*_ + 1)∕2⌋) and final (⌊(τ_post_ − τ_pre_) *f*_*s*_⌋) frames of the cut-out interval, to avoid the high amplitudes of the spike itself.

*Amplitudes on individual channels A*_*i*_. The (inverted) signal was convolved with a kernel ($\frac{1}{6},\frac{1}{3},\frac{1}{3},\frac{1}{6}$) of 4 frames length (cutoff frequency 0.14 *f*_*s*_, to account for smaller, low-pass filtered amplitudes on more distant electrodes) and its maximum around the detected peak time *t*_0_ was determined.

*Boundaries and outliers*. Amplitudes of channels where signals were missing due to the chip boundary or voltage drifts out of the linear regime of amplifiers, would induce a bias in the location estimate if they were set to zero. To reduce that bias, we counted how many of the eight adjacent channels were available and used the product of their number with 1/8 of the median of their amplitudes as a surrogate signal. To further reduce the effect of outliers, we restricted amplitudes to positive values and subtracted the 20th percentile of their values. Except for the center channel, we reduced amplitudes that exceeded the sum of their eight neighboring amplitudes for channels used in the detection and 2/3 of that sum for other channels. For that, amplitudes were clipped to 1/2 or 1/3 of the sum over amplitudes in a $\sqrt{2}d_{\text{pitch}}$ (i.e., nine channel) neighborhood, respectively.

*Localization*. Finally, the amplitude median was subtracted, amplitudes were restricted to positive values, and the barycenter $\hat{B}$ of the peak amplitudes was determined. As nearby spikes would interfere with the signal, we multiplicatively reduced the amplitudes around the peak location such that amplitudes for distances between *d*_pitch_ and 2 *d*_pitch_ would linearly drop to zero. Then we determined a new center of mass and subsequently performed the distance dependent weighting again, this time weighting amplitudes beyond 2 *d*_pitch_ negatively (clipped at –0.1). The center of mass of these amplitudes was used as a final location.

*Amplitudes* Â. We used half of the sum of amplitudes after the localization step for further analysis. This was done as we expected the center electrode to contribute about half of that sum. It underestimated true signal amplitudes due to the subtraction of the spatial median and since we performed a temporal averaging as well, but reduced noise.

### 2.4. Events of similar spatio-temporal origin

The spike detection algorithm removed events in the refractory period (Hill et al., 2011) if spikes appeared on the same electrode by finding the largest amplitude event within a temporal window of 1 ms. However, the same spike could still be detected on different electrodes and assigned a similar location. To avoid duplicates, we only retained the event with the highest amplitude in a spatio-temporal neighborhood.

*Online detection*. Events that were detected with a higher amplitude within 0.5 ms before and after the event within a distance of 60 µm were removed, as these were likely caused by the same signal source. The fraction of such events was only about 5% in a recording from a culture, but would be as high as 50% in this retina recording, where the observed spike amplitudes were generally higher.

*Interpolating detection*. We removed events if there was a higher event within 0.5 ms before and after the event and within a distance of 42 µm.

### 2.5. Synthetic data

The exact measured spike shapes depended on the neuron, the conductivity of the media etc. and were undersampled both in time and space such that a direct analysis of detection performance and spatial biases using measured spike templates was not feasible. Neither it was desirable, as it would be specific to the preparation and therefore not suitable to systematically characterize the detection performance. Instead we defined a spike-like voltage trace (distorted sine with zero mean as shown in **Figure 3A**) and weakened the amplitude as a function of distance from an effective electrode boundary.

The signal attenuation with distance depends on the local conductivity and distance from the electrode surface and therefore varied within a single preparation. We considered a special case, where the electrodes were given an effective spatial area of 16.8 × 16.8 µm^2^ where current sources would be detected with equal amplitudes. The minimum resistance between the current source and an electrode was modeled by an offset distance *r*_*offset*_ of *d*_*pitch*_∕5 = 8.4 µm. Outside the effective electrode area, we assumed the potential to fall off as ~ 1∕*r* with distance *r*.

A signal with amplitude *a*_0_ at horizontal distances *r*_*x*_ and *r*_*y*_ from the center of an electrode would then be measured with an amplitude *a* given by the following equation, where Θ denotes the Heaviside step function:

a=a0 roffset((rx-roffset)2Θ(rx-roffset)+(ry-roffset)2          Θ(ry-roffset)+roffset).

Such waveforms were generated for 16 different positions with respect to the closest electrode and sampled with 16 different timelags ($\Delta t={\left(16f_{s}\right)}^{-1}$). Those were inserted into 256 patches of 3 × 3 electrodes of a recording from an empty chip, spaced by four electrodes spatially and by 256/*f*_*s*_ temporally, using 15 different amplitudes for each patch. The spike detection and localization was performed and the results compared to the injected events.

For comparison, we also implemented a conventional threshold-based detection, where the threshold for each channel was set to a multiple of the noise variance (estimated without inserting spikes). Data were band-pass filtered using a 0.1–2 kHz 3-pole Bessel filter.

Detected events were assigned to inserted events if they occured within 3∕*f*_*s*_ and a radius of $\sqrt{2}∕2d_{pitch}$ of the inserted event. If multiple events were found in that range, the temporally closer event was used. Receiver operating characteristic (ROC) curves for each channel were obtained by sorting amplitudes of false positives, determining thresholds for different false positive rates and the corresponding true positive rates. To then summarize a range of different conditions (**Figure 3D**), we quantified the detection performance as the area under ROC curves up to a false positive rate of 0.1 Hz since the detection was performed continuously in time and higher false positive rates would not be relevant.

For the interpolating method, we further computed the median location for each of the 256 patches and three amplitude ranges for the inserted spikes. The median of the distances between the estimated and inserted locations of events was determined as error in the localization. Part of this distance was due to a systematic bias, quantified as the distance between the median location of the detected events and the inserted location.

Using different parameters for the offset, effective electrode size and attenuation scale did not qualitatively change the results (not shown). We did not attempt to employ measured templates for this analysis as they could not be shifted systematically in space due to the spatial undersampling.

### 2.6. Correlation analysis

This analysis had several aims, first to estimate the amount of spikes lost due to a poor SNR, second to determine an appropriate detection threshold, and third to validate the detection method. The method exploited the fact that spikes in our preparations were typically correlated, at least weakly, due to network interactions, while electrode noise was uncorrelated. Therefore, the presence of significant correlations should be indicative of true spikes while uncorrelated events would more likely reflect recording noise. We note that while this was a reasonable assumption for the preparations used here, it obviously does not generally apply.

Here, we first provide a motivation for the analysis steps, before explaining each step in detail.

Technically, the quantification of correlated activity was challenging since the measured activity was highly non-stationary and typically sparse on most of the electrodes. Additionally, we aimed to infer the fraction of events participating in correlated activity rather than a correlation strength, such that we needed to distinguish single events.

In short, these challenges were addressed as follows:

1. The temporal order of spike times (rank) was used to measure temporal differences.
2. Pairwise correlations between channels were determined across the chip.
3. Coactive, correlated channels determined a correlation index for each event.
4. The distributions of correlation indices of measured and surrogate random events were compared.

#### 2.6.1. Ranking spike times

Due to the sparseness of neural activity measures such as Pearson correlation coefficient were not very informative of interactions between neurons, but rather reflected the synchronous lack of spiking. In addition, Ventura and Gerkin (2012) showed that even misclassification of events lead to a bias in correlation estimates unless the inter-event intervals were independent and Poisson distributed. We were interested in finding more direct interactions, and to, at least partially, compensate for effects of fluctuating firing rates and reduce the regularity of inter-event intervals. In order to speed up the analysis and reduce memory requirements, we selected a number of reference units (showing high activity) and correlated their activity with the activity seen at each location (including reference units) across the whole recording.

*Online detection*. The firing rate of each channel was compared to channels within a distance of 42 µm. Channels with a local maximum in firing rate were defined as reference channels.

*Interpolating detection*. We divided locations of events in a raster of 192 × 192 bins to increase the spatial resolution by a factor of three. This resolution is sufficient to capture more than 80% of the area of any spot of activity with a diameter of *d*_*pitch*_ in a subset of 3 × 3 bins. In order to obtain a sufficient sample size to estimate correlations and for a comparison to the online detection results, we then used events from every 3 × 3 bins as units for further analysis. For choosing reference units, we used a finer binning (320 × 320 bins) to determine spatial maxima in the event density. To this end, we smoothened the histogram (distance dependent, cone shaped kernel with a radius of 15 µm), and detected local maxima within a radius of 15 µm. This was done to ensure that they are not originating from the same current source and to separate closely adjacent maxima. We used events from bins within a radius of 21 µm from those maxima as reference units. Overlapping bins were assigned to the closer maximum.

To minimize spurious effects due to very noisy channels, the 10 (parameter, adjustable) most active reference units were excluded as they might be noisy. As surrogate data, random (Poissonian in population rank) events were added at a rate of 0.1 Hz (as parameter, adjustable) per channel. This preserved relative changes in population rate. We did not add this noise into the reference units. Additionally a clock unit firing every fourth frame (0.57 ms) was added (to the reference units) to establish a maximum time window in which correlations were computed. Then all (reference) events were ranked across the whole chip, and the event rank was used as a new time axis.

For each unit *i*, a histogram of all reference units *j* coactive within a temporal τ_corr_∕2 event neighborhood was collected. For an independent Poisson process, the number of such coincidences follows a Poisson distribution, and its mean can be estimated from the marginal distribution of such coincidences (around any spike) divided by the total number of events (including Poisson events) and multiplied with the number of events in the channel:

$${\hat{n}}_{ij}=\frac{\tau_{\text{corr}}n_{i}n_{j}}{\sum_{k}n_{k}},$$

where *n*_*k*_ denotes the total spike count in channel *k*.

Channels with very different activity patterns would increase $\underset{k}{\sum }n_{k}$, but barely affect the number of coincidences. As a result, units could appear as correlated only because they were not correlated with a subset of units that showed an entirely different behavior. In our case, noise often resembled a Poisson process whereas activity occurred in bursts. To avoid classifying activity of the same kind as correlated, links between pairs of channels with significantly fewer (*p* < 10^−6^) coincident spikes than expected by random were removed from the statistics. Similarly, we only compared units to reference units with a distance larger than 42 µm. For the online detection, we did not compare directly adjacent channels. Finally, all pairs of channels with reference channels [*i, j*] with more coincident spikes than expected (*p* > 0.1) were identified and stored in a boolean matrix (*A*_*ij*_ = 1).

#### 2.6.2. Correlation index

Knowing which channels have correlated activity, it was possible to decide for each spike individually to what extent it participated in the network activity. We defined a correlation index (CI) as the fraction of spikes within a temporal τ_corr_∕2 event neighborhood that came from correlated channels multiplied by the fraction of significant correlations among those channels. Depending on the preparation, this method could be further refined, for instance by computing the CI separately for different temporal or spatial windows and using the maximum of these estimates. An example of the correlation index distribution is shown in **Figure 5A**.

#### 2.6.3. Fraction of uncorrelated events

For each channel *j* the detected events were sorted according to the correlation index and signal amplitudes (permutation operators σ_*CI*_ and σ_*Amp*_). We then determined the empirical cumulative distribution of the correlation indices of the inserted Poisson events ${\hat{F}}_{Poisson}\left(CI\right)$ and determined its values at the correlation indices of the detected events. Event amplitudes were ranked to obtain a measure *r*_*Amp*_ that did not depend on the amplitude range. Then the following quantity was computed:

$$\begin{array}{l} X_{k}=\frac{1}{2N_{j}}\overset{k}{\underset{i=0}{\sum }}r_{Amp}\left(\sigma_{CI}\left(i\right)\right)+\frac{1}{2}\frac{\sum_{i=0}^{k}{\hat{F}}_{Poisson}\left(CI\left(\sigma_{Amp}\left(i\right)\right)\right)}{\sum_{i=0}^{N_{j}}{\hat{F}}_{Poisson}\left(CI\left(i\right)\right)}\\ k\in \left\{1,\ldots ,N_{j}\right\}, \end{array}$$

where *N*_*j*_ is the number of detected events and *CI* denotes the correlation index. For independent correlation indices and amplitudes, *X* would be the empirical cumulative distribution of a uniform distribution. The deviation from the cumulative uniform distribution is shown in **Figure 5B** for a recording from a culture. For samples where the distributions of inserted Poisson and detected events are equal, this deviation is described by a Brownian bridge which has its maximum variance at *N*_*j*_∕2.

We tested against a uniform distribution with a one-sided Kolmogorov Smirnov (KS) test (*p* < 0.01) (see **Figure 5C**). For channels that passed this test, we normalized this deviation by dividing by the standard deviation of the corresponding Brownian bridge $2\sqrt{k\left(N_{j}-k\right)}∕N_{j}$ to avoid a statistical bias toward *N*_*j*_∕2. The argument of the maximum deviation $P_{j}={argmax}_{k}\left(\left(X_{k}-k∕N_{j}\right)\left(N_{j}∕2\sqrt{k\left(N_{j}-k\right)}\right)\right)$ then determined the boundary between correlated and uncorrelated events.

For channels where the KS test was not significant, we computed the fraction of inserted Poisson spikes with smaller CI for each event, subtracted this fraction and divided by the fraction of events with larger CI. This quantity was clipped to [0, 1] and averaged for all events and used as an estimate of the fraction of correlated events. If the CI distributions of detected events and Poisson spikes were the same, this number was 0, and it was only 1 if they were not overlapping. Hence this was potentially a rather conservative measure of correlated activity.

#### 2.6.4. Extension to single spikes

In order to obtain a per event probability for correlated events, the correlation indices were sorted for both the detected events and inserted Poisson events of each channel.

The fraction of events *f*_*CI*_ was computed in a sliding window, using reflective boundaries:

$$f_{CI,k}=\overset{q\left(k\right)+L}{\underset{i=q\left(k\right)-L}{\sum }}\frac{x\left(\sigma_{CI}\left(i\right)\right)}{2L}\;k\in \left\{1,\ldots ,N_{j}\right\},$$

where *x* is a boolean vector distinguishing real and Poisson events, σ_*CI*_ a permutation operator that sorts according to correlation index, and *q* a vector of the indices of real events. We optimized the length 2*L* of that sliding window such that its length would be minimal and the fraction of false events after renormalizing would not exceed 1.1. For channels with a high fraction of noise, that would bias toward using a large window. Especially for a strong relation between the correlation index and the fraction of Poisson events, small windows would be used.

This procedure mapped correlation indices to the fraction of Poisson events and needed to be normalized to match the fraction of uncorrelated events determined for each channel

$$p_{Spikes,k,j}=\frac{f_{CI,k}\cdot P_{j}}{\sum_{k}f_{CI,k}}.$$

This fraction then determined the probability that an event should be classified as uncorrelated.

### 2.7. Spatial activity profiles

Here we aimed at visualizing relations between temporally adjacent events in two-dimensional, high resolution maps. To this end, for each event the relative angles of all events within a defined spatial range (82 µm < × < 1092 µm) and time window (± 30 event ranks) were collected and spatially averaged using a spatial sliding window (17.5 µm wide). This provided an estimate of the direction toward the center of mass of coincident activity. This was strongly biased toward the center of the chip, where the average activity was centered.

To then identify local structures, which characterize network activity, an average computed over a large sliding window (336 × 336 µm^2^) was subtracted from this density (**Figure 9**). As this procedure would correspond to a random walk for random spikes, the density plot was normalized by $1∕\left(3\sqrt{N}\right)$, such that a pure color now corresponded to three standard deviations different from random, and gray indicated random behavior (**Figure 9C**, right panel).

## 3. Results

We used recordings from cultured hippocampal neurons and from the spontaneously active perinatal retina. In addition, recordings from an empty chip and a pharmacologically silenced retina were used as control, and to generate synthetic data for validation. Here we first introduce two methods for spike detection. The first works independently on single channels, is highly efficient and online-capable, and can be used on any type of electrical recording. This method was then extended to make use of signals from the same spike in multiple electrodes to improve SNR, and to localize event source locations.

Next, we assessed spike detection and localization performance by analysis of synthetic data. As further independent validation we compared estimated spike locations to the physical positions of neurons on the array in an anatomical micrograph, and estimated the detection performance on real data by exploiting correlations in neural activity. Finally, we examined the possibility to directly analyze spatio-temporal network activity profiles of a neural culture, where clustering into single units was not always feasible.

### 3.1. Separate treatment of channels allowed to detect spikes online

We discuss results for the online detection first, and then highlight differences for the extension where signal interpolation was used, as the subsequent analysis performed is similar in both cases. Here we provide an informal description of the methods, technical details, and parameters are given in the Section 2 and Table 1.

Our aim was to detect significant negative deflections of the recorded voltage from baseline (Figure 1A), which required a reliable estimate of the baseline and noise amplitude. Baseline fluctuations were frequent, and the noise had a strongly non-Gaussian form (Figures 1B,C; Fee et al., 1996), making a simple variance estimate unsuitable. Therefore, similar to Rossant et al. (2015) we used continuously updated measures based on signal quantiles to estimate noise. Specifically, an online estimate of the 33rd percentile of the voltage served as baseline and an adapting variability estimate *v* served to define the detection threshold (Figures 1D,E; see Section 2 for details).

This estimate was purposefully made asymmetric because of the non-Gaussian noise, to estimate the voltage where the probability density drops significantly. Percentiles were estimated instead of performing a high-pass filtering step as large deviations from baseline would otherwise leak into neighboring recording frames, reducing the SNR. This problem was addressed by cutting out large fluctuations (Prentice et al., 2011; Marre et al., 2012), but this requires an additional threshold and is computationally more demanding. The variability estimate used here is specific to fluctuations in the negative direction and again based on percentile estimates.

Events exceeding the threshold were then accepted as spikes, subject to additional adjustable requirements for their duration and repolarization. Moreover, simultaneous events in neighboring electrodes were assigned to the electrode with the strongest signal to prevent detection of the same neuron multiple times.

This method allowed for a quick assessment of the recorded activity, and formed the basis for the interpolating method described next.

### 3.2. Interpolation improved the spatial resolution of MEAs

As a next step, we extended this detection algorithm to exploit the fact that typically signals from the same neuron could be found on multiple adjacent channels. In particular in the perinatal retina we often recorded spikes with large amplitudes (several hundreds of µV), and at the same time, smaller, synchronous events were frequently detected on neighboring electrodes. In cultures, the effective conductivity of the growth medium was higher due to the lower density of cells, leading to smaller but more widespread signals. As many spikes had peak amplitudes close to the detection threshold, the spikes detected on each electrode separately were likely only an incomplete record of the activity of a single neuron, in particular if it was located between electrodes.

Therefore, combining the signals of multiple, nearby electrodes before performing event detection would reduce noise and prevent undesired separation of events. If the spatio-temporal profile of a spike was known and the noise was independent and Gaussian, a dot product of a template with the signals could be employed to identify spikes (Marre et al., 2012). In order to avoid estimating exact templates, we assumed that the largest current source has a small spatial extent compared to the electrode spacing *d*_*pitch*_ and only distinguished between events with sources either close to or between recording electrodes. Of course, detecting events for both cases effectively constituted multiple testing, but these tests were for different spatial positions on the array and thus could still be distinguished *post-hoc*.

**Figure 2:**
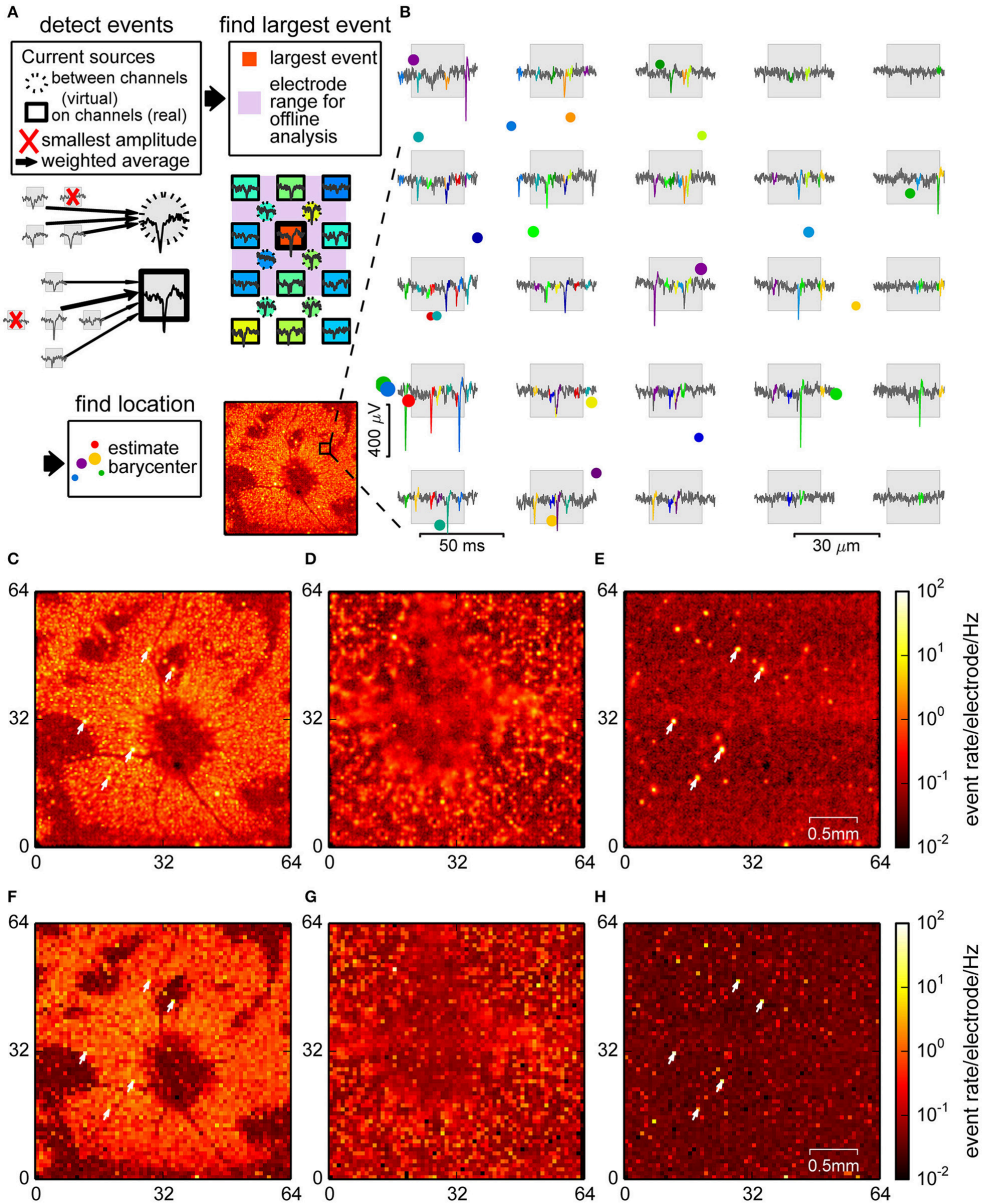
**Spike detection through signal interpolation**. **(A)** Illustration of the interpolating spike detection. Voltage traces from groups of 4 and 5 channels were averaged, and local maxima were used to determine electrode ranges for offline localization (insets). **(B)** Example of detected spikes at their estimated locations (colored dots, size relates to event amplitude), and the corresponding events in the raw data from nearby channels. **(C–E)** Spatial density plot of events detected in the retina **(C)**, culture **(D)**, and empty chip **(E)** recording. **(F–H)** Corresponding plots for the online detection method. Recordings shown in **(C,E,F)** and **(H)** were performed with the same chip. Arrowheads point to noisy channels. Parts **(A,B)** show data from a retina.

Specifically, the signals of five channels, one in the center and four surrounding, were used for signals that mostly occurred on one electrode. Blocks of 2 × 2 channels were used for signal sources between electrodes. In both cases, the lowest signal amplitude was ignored (Figure 2A). Spike detection was then performed as above, but on weighted signals. Optimal weights depend non-linearly on the inter-electrode distance of the hardware but may be approximated after measuring the spatial decay of spike amplitudes (Supplementary Figure 1). This weighting partially allowed compensating for the weaker signals expected from neurons further away from recording channels.

For each event the center of mass of the spatial distribution of spike amplitudes was used to estimate a putative event source location (see Figure 2B for examples and Section 2 for details). This method was indeed capable of revealing structures at a higher spatial resolution, as visualized in the spike count histograms in Figures 2C,D. In the retina (Figure 2C), events were strongly clustered, with several elongated areas without spikes, most likely corresponding to blood vessels, which were less conspicuous in the online method (Figure 2F). In the neural culture (Figure 2D), many areas had high spike densities, where small local clusters that may have originated from single neurons could not be resolved. The empty chip recording (Figure 2E), in contrast, showed a largely uniform background with a low density of falsely detected events, and a few highly localized clusters which corresponded to noisy channels.

### 3.3. Synthetic data revealed that interpolation sharpens the detection threshold

**Figure 3:**
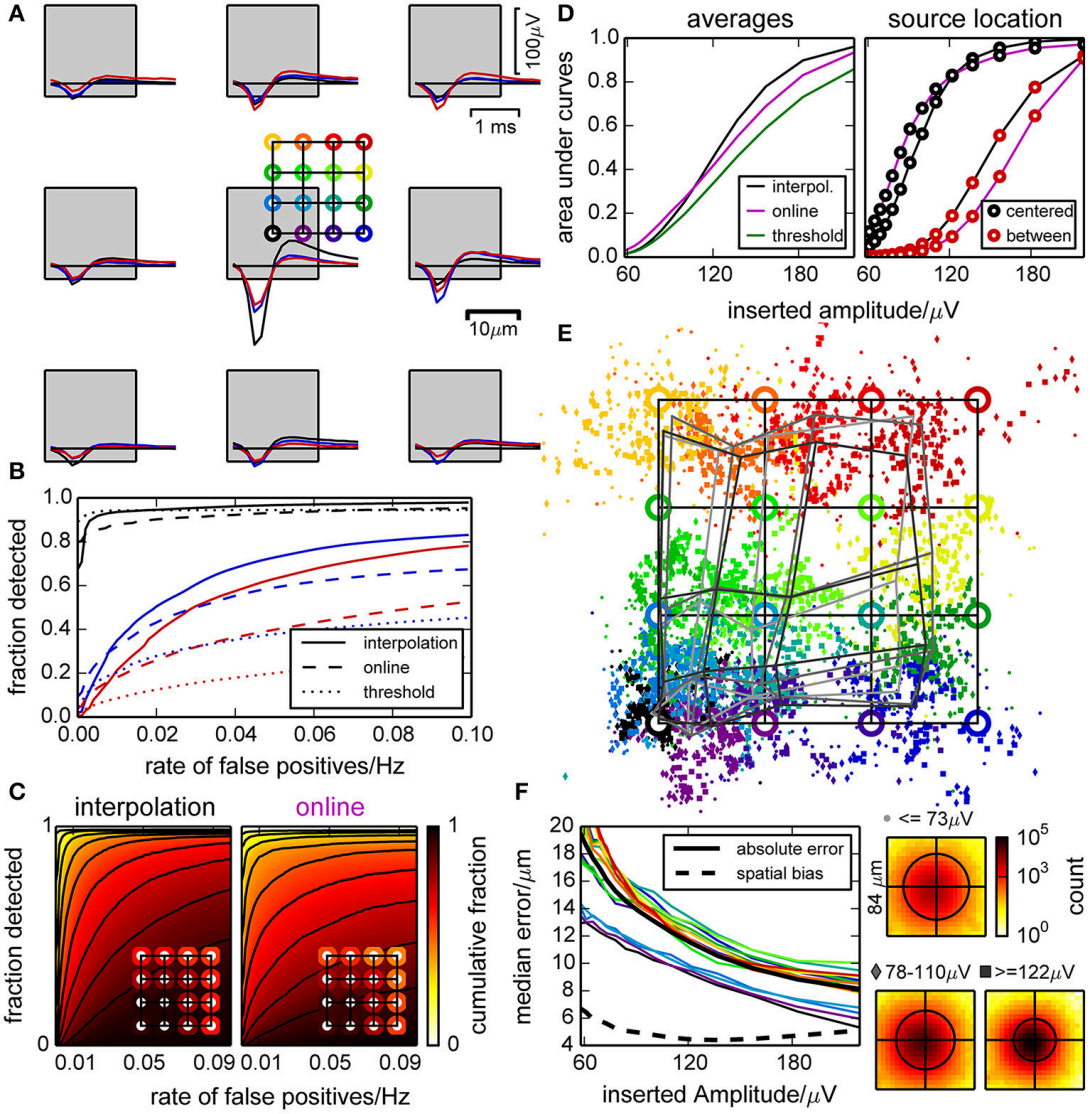
**Performance of the spike detection and localization evaluated with synthetic data**. **(A)** Artificial spikes were inserted in a recording from an empty chip with realistic noise profile at positions indicated by the colored open circles. This defines a color code that is used in panels **(A,B)** and **(D–F)**. Squares indicate the electrode locations and dimensions. **(B)** Example ROC curves for inserted spikes as shown in **(A)**, constructed by varying the detection threshold. Positions are indicated by color, and curves are shown for the online and interpolating methods and a simple constant threshold. **(C)** Illustration of the variability in detection performance for spikes inserted near different electrodes, at 16 different positions relative to those electrodes [as in **(A)**] and with five different amplitudes (>120 µV). The plots show in how many cases the detection performance (“fraction detected”) was at least as high as indicated by color. The insets illustrate the average fractions of detected spikes (i.e., averaged color) for different positions of the inserted spikes. **(D)** Performance of the detection methods, represented as the average area under ROC curves with respect to the inserted spike amplitudes (left). For specific locations of inserted spikes, we observed a large variability [right, cf. definition of circle colors in **(A)**]. **(E)** Median positions of detected spikes with respect to their theoretical (specified) positions (circles). Due to the clipping of the signal at its median and a biased normalization factor (due to noise) when computing the barycenter, events tend to be found closer to electrodes. The gray grids illustrate the median distortion at different amplitudes. **(F)** Median absolute shift of detected spikes for each inserted location, and median for all (thick black line). Also shown is the median absolute shift from the median detected locations (dashed black line). Insets show histograms of positions of all detected spikes of a given amplitude with respect to the inserted position (black circles illustrate 90 percentiles).

To evaluate the detection performance of our algorithm and the precision of spatial event localization, we created templates for artificial spikes with different amplitudes, current source locations with respect to the closest electrode (illustrated in Figure 3A) and time lags with respect to the sampling. Those were added to the raw traces of the empty chip recording, and spike detection and localization was performed.

Spikes were detected most efficiently when they originated close to an electrode, and a peak amplitude of 120–160 µV was required for a reliable detection. We determined receiver operating characteristic ROC curves for different electrodes, spatial offsets, and amplitudes of inserted spikes (examples shown in Figure 3B, distributions in Figure 3C). As there was a strong dependency on the amplitudes of inserted spikes, we restricted this analysis to amplitudes larger than 120 µV. Further, we averaged the ROC curves for the same amplitude and false positive range for individual insertion locations (insets). This revealed a large variability in detection quality across electrodes and current source locations for all detection methods used here. In case of the online and thresholding approach, spikes with small amplitudes could not be detected at all locations.

A systematic comparison across amplitudes is shown in Figure 3D. We observed that the online detection method did perform better with low signal amplitudes, but this would lead to an incomplete spike record and is therefore not desirable. The interpolation method had the steepest slope for an increasing amplitude and hence discriminated best between different amplitudes. Additionally the dependence on the insertion location was less pronounced for the interpolating method. Detecting spikes by simple thresholding did not yield satisfying results.

The spatial spread of detected events from the same source location decreased with increasing amplitude, but for locations away from an electrode estimates were biased toward the electrode center (Figure 3E). This distorted the image of current source locations, and the distortion barely decreased with signal amplitude (Figure 3F). This bias was due to the use of signal amplitudes clipped at their median to estimate event locations. For high signal amplitudes, the amplitudes of the surrounding electrodes became more reliable and hence the median was a biased estimate of the baseline. For small amplitudes, however, this increased baseline estimate was useful as it reduced the influence of noise on the localization. Thus, in order to reduce the uncertainty of the location estimate, a bias was unavoidable. For a reliable classification of spikes based on location, it would however not be useful to obtain statistically unbiased locations if the spatial spread increased more than the reduction in bias. Therefore, we optimized the method to obtain a good compromise between the number of channels used for localization, and the level of noise that entered this estimate.

In summary, this analysis showed that the performance of the detection of artificial spikes did depend on the position of the current source. Moreover, the location estimate was biased, but retaining this bias reduced the spatial jitter such that different sources could be distinguished at a resolution finer than the spacing of the electrodes. Signal interpolation yielded, as expected, an improved detection performance compared to the online method.

### 3.4. Somata and hotspots of activity were found in apposition

**Figure 4:**
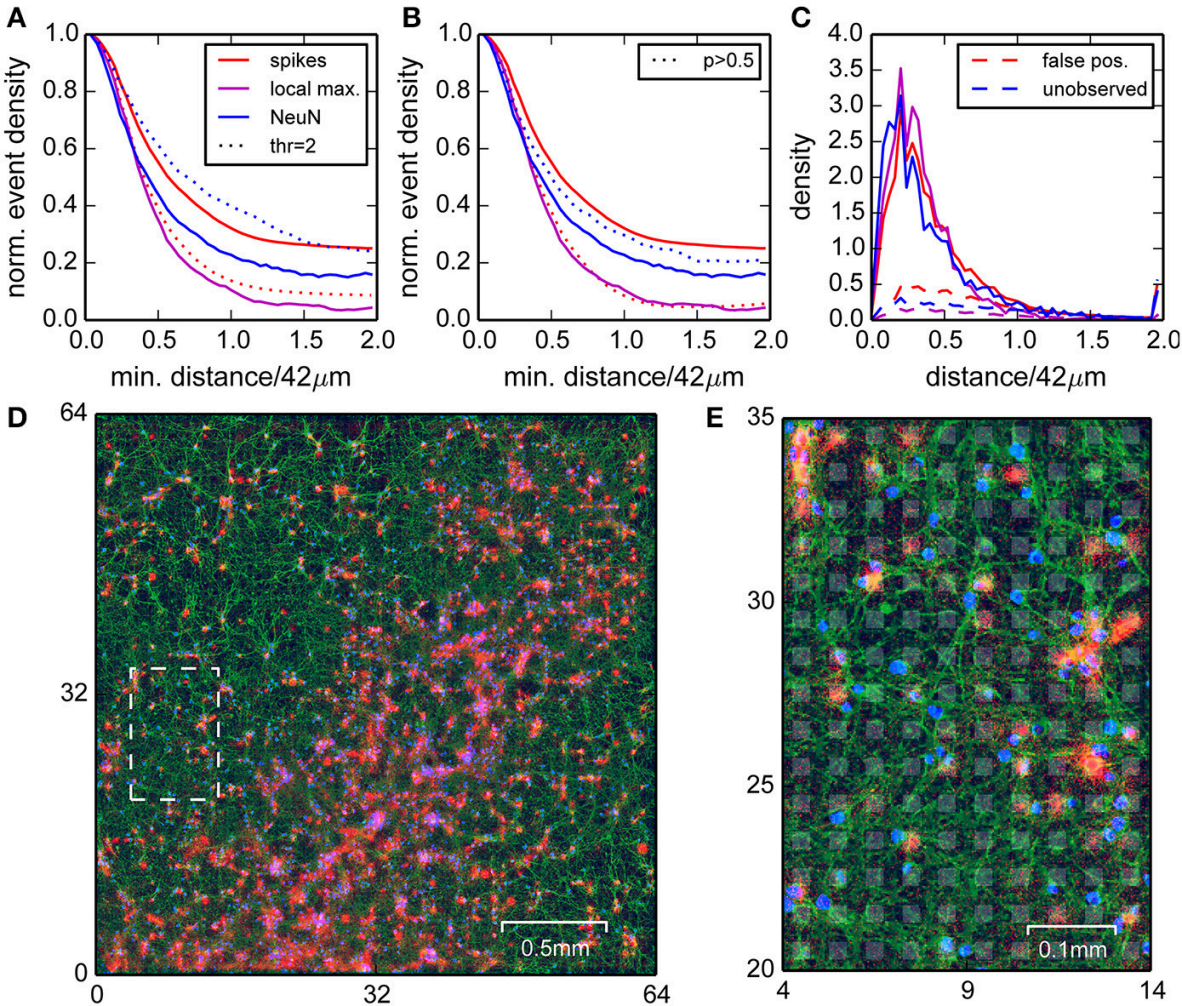
**Comparison of positions of neural nuclei with the locations of detected events**. **(A)** Normalized densities of events (red) and local maxima of the spike density (magenta) when restricting to areas with a minimum distance to the nearest neural nuclei. Blue curves are normalized densities of neural nuclei after restricting to areas with a minimum distance to the nearest local maximum in the spike density. Dotted lines correspond to a higher detection threshold. **(B)** as in **(A)**, but with dotted lines corresponding to correlated events with probability *p* > 0.5 (see Section *Events Found on Noisy Channels did not Participate in Correlated Activity*). **(C)** Densities for distances of spikes (red, solid lines), local maxima (magenta), and neural nuclei (blue) compared to densities obtained for homogeneous distributions (normalized to minimum density around 42 µm, dashed lines). **(D)** Image of the culture with NeuN (blue), tubulin staining (green), and the detected spikes (red). A close-up of the region indicated by the dashed white box is shown in **(E)**. Electrode locations are depicted as gray background patches.

To investigate whether the detected and localized spikes indeed originated from neurons, we stained a sparsely seeded culture with fluorescent antibodies for beta tubulin III and neural nuclei (NeuN) after the recording (Ullo et al., 2014), and then compared the estimated spike locations in the recording with the actual locations of neurons (Figure 4). A sparse culture was used because it allowed a clear visual separation of somata, which was more difficult in the retina or dense cultures. The tubulin staining revealed that neurons covered the entire chip, and thus electrical activity could in principle be recorded everywhere. However, the strongest signals were expected near neural somata, where spikes were initiated at the axon initial segment.

The NeuN staining showed that the somata of neurons were inhomogeneously distributed, which allowed for direct comparison between estimated spike and cell locations. We assumed that falsely detected spikes were uniformly distributed, and true spikes should be detected close to a nucleus. We began this analysis with a low detection threshold, where we expected a better detection of small events, but also a higher number of false positives. To confirm this intuition, we computed the normalized spike density found at least a distance *x* away from a nucleus, and varied the distance *x* from 0 to 84 µm (Figure 4A, red line). This showed that this density dropped to a constant 25% for distances larger than 60 µm, hence around 75% of all detected events were close to one of the labeled nuclei and therefore likely true spikes.

The reverse analysis, i.e., measuring spike distances from nuclei, showed that around 80% of the nuclei had a peak in spike density within a radius of 40 µm (Figure 4A, blue line). Moreover, about 90% of the peaks in spike density had a corresponding nucleus within 40 µm (Figure 4A, magenta line). Increasing the detection threshold increased the fraction of spikes detected close to a nucleus (Figure 4A, dotted red line), but also reduced the fraction of nuclei with associated local maximum (Figure 4A, dotted blue line), hence improved the rejection of false positives, but also increased the number of false negatives.

These findings were further confirmed by considering the corresponding densities of spikes and nuclei as a function of distance from nuclei and spikes, respectively (Figure 4C, solid lines). These distributions always sharply peaked at around 10 µm, close to the distance of the axon initial segments of the neurons. Importantly, these densities differed substantially from surrogate data from homogeneous distributions (Figure 4C, dashed lines).

In summary, we showed that high amplitude events were mostly detected close to neuronal somata, but not all somata could be associated with a cluster of events. For some somata, a corresponding cluster of events could be identified when lowering the detection threshold, suggesting that they were indeed active, but weakly coupled to the chip.

### 3.5. Events found on noisy channels did not participate in correlated activity

Since correlations in neural activity resulted from network interactions, we assumed that detected events with significant correlations with other events were most likely true spikes, while the events due to electrode noise should always be uncorrelated. In particular, when a parameter change lead to an increase in the number of detected spikes with significant correlations, we could relate this directly to improved detection. On the other hand, we obviously could not assume that all uncorrelated events were caused by noise, so these only provided an upper bound for the number of falsely detected events.

**Figure 5:**
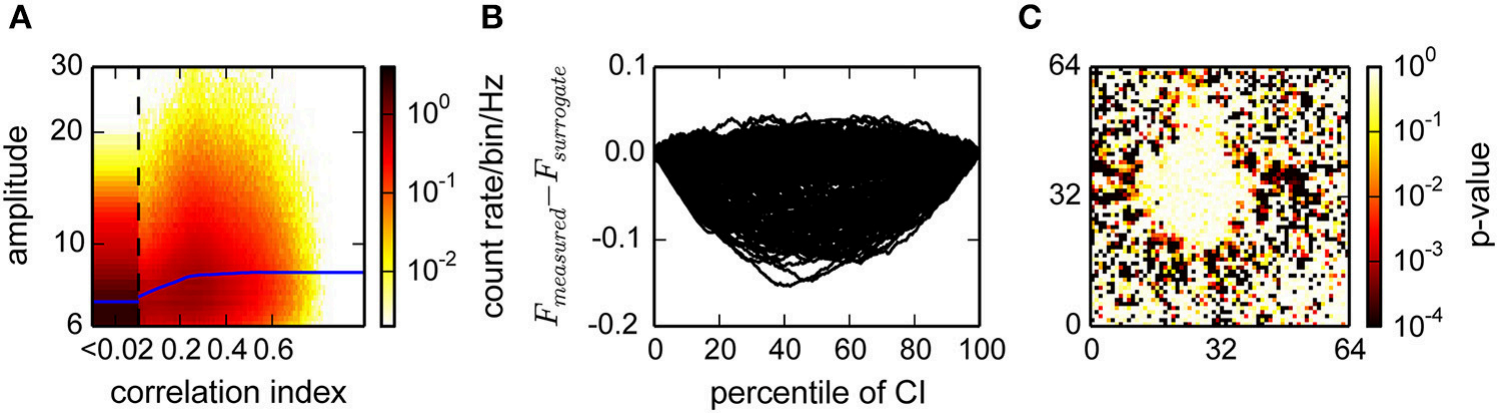
**Estimation of the fraction of uncorrelated events in a recording from cultured neurons**. **(A)** Histogram of the joint distribution of amplitudes, expressed as multiples of the variability estimate *v*, and correlation indices. The first bin (CI < 0.02) was rescaled by 1/20 and widened accordingly. Blue lines represent a sliding median of the amplitudes. **(B)** Difference between the distributions of measured and surrogate events. The graph shows the Kolmogorov Smirnoff test statistic for a random subset of electrodes from the data in **(A)**. Negative values indicate a larger fraction of surrogate events with a lower correlation index. Events with a correlation index higher than the argument of the minimum of those curves are classified as correlated. **(C)** *p*-values for deviation from identical distributions computed as illustrated in **(B)**, for all channels. Red colors indicate that a channel is highly unlikely to be reporting noise.

As the activity in cultures was very non-stationary, which would bias a temporal correlation estimate during periods of high or low activity, we considered a temporal neighborhood of a fixed number of events rather than a fixed time interval. Our strategy was to then count how many of these events are from units with correlated activity. This was quantified using the correlation index (CI), which is related to its propensity to participate in correlated activity (see Section 2). The CI was at least weakly dependent on the amplitude of the events, indicating that it provided an estimate of true spikes (Figure 5A). For statistical analysis, we next compared the CI distribution for each unit to the distribution obtained from randomly inserted events by computing the difference of the respective cumulative distributions (Figure 5B). Units with a different average CI (KS-test, Figure 5C) could then be separated into a correlated and uncorrelated fraction (Minima in Figure 5B).

**Figure 6:**
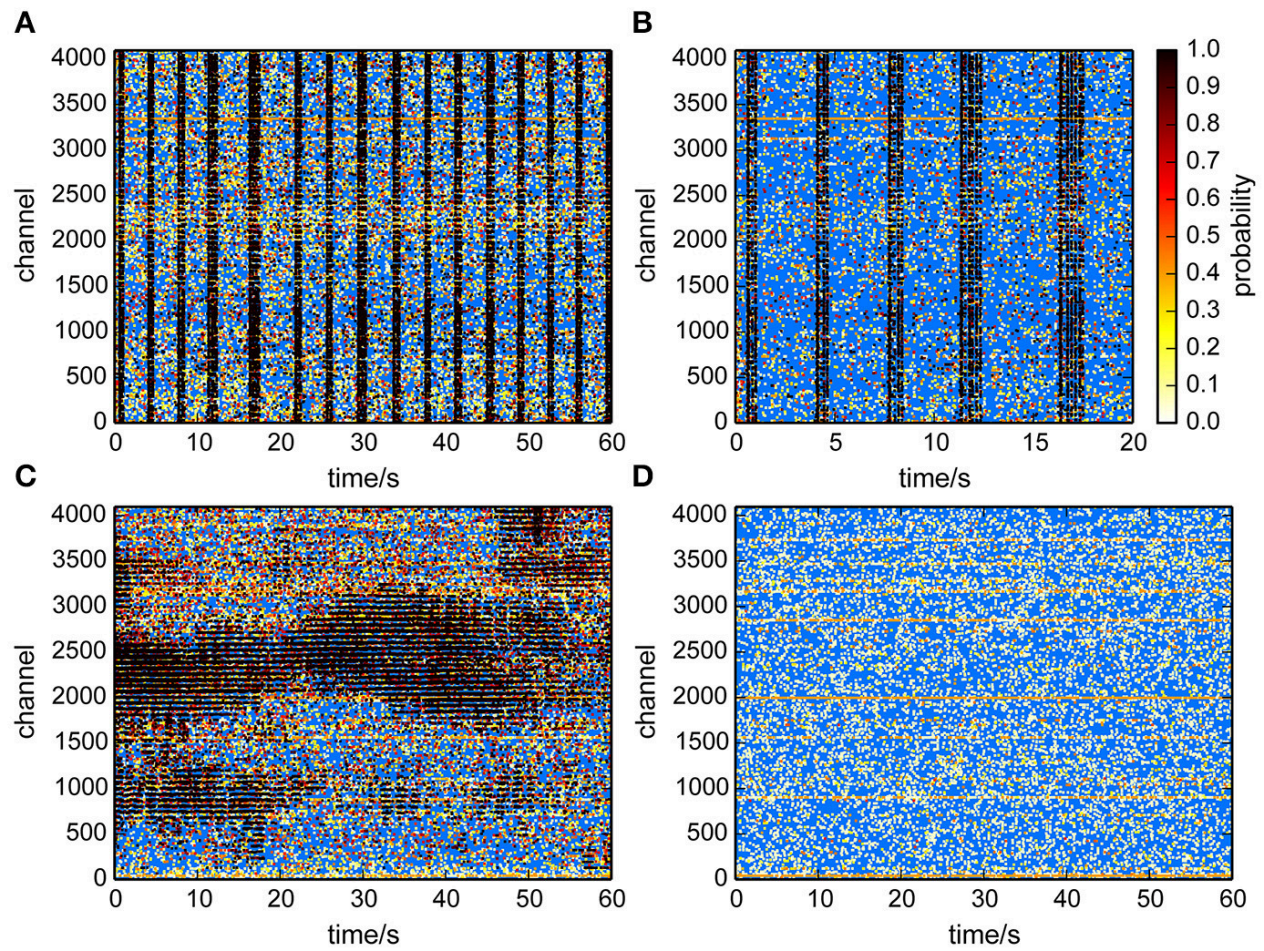
**Raster plots with probabilities that individual events participated in correlated activity**. A high probability (black dots) indicates that an event likely reflected neural activity. **(A,B)** Culture recordings. **(B)** is same data as in **(A)** but for a shorter time-window. **(C)** Retina recording. Correlated activity was found within retinal waves. **(D)** Empty chip recording. Events were classified as uncorrelated.

This method provided an estimate of the number of uncorrelated events per channel, and assigned each event a probability to participate in correlated network activity. This was visualized in raster plots (Figure 6) for recordings from cultured neurons (Figures 6A,B) and a retina (Figure 6C). Each putative spike was represented as a dot, where the color indicated the probability that it was a correlated event, hence a true spike. It was clearly apparent that events with a high probability primarily occured during population burst events. Application of the same algorithm to a recording from an empty MEA confirmed that recording noise was indeed uncorrelated and therefore assigned low probabilities to virtually all detected events (Figure 6D).

**Figure 7:**
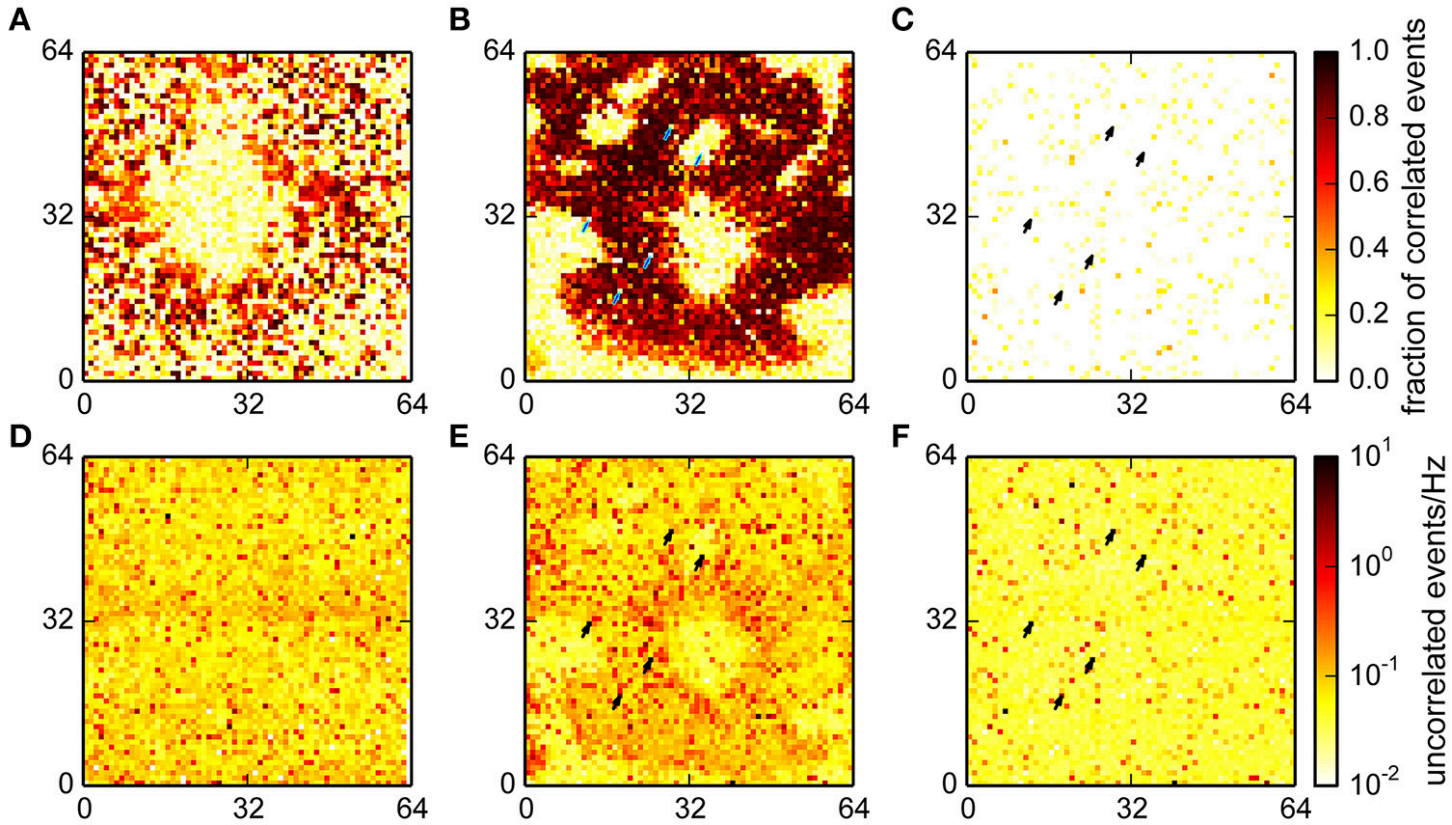
**Fraction of events classified as correlated for a culture (A), retina (B) and empty chip (C) recording and the corresponding rates for uncorrelated events (D–F)**. A high fraction of correlated events indicates the presence of a neuron that is participating in network activity. More uncorrelated events were found in the retina recording in an area with good electrical coupling where the total number of detected events was an order of magnitude higher. Note that for the retina and the empty chip recording, the same chip was used, and the same channels were detected as noisy (arrows).

The spatial distributions of the fraction of correlated and number of uncorrelated events in the different recordings are shown in Figure 7. In recordings from cultured neurons, correlated events were spatially clustered, which reflects the tendency of neurons to forms small, interconnected groups. Uncorrelated events were mostly uniformly distributed in this recording, as may be expected from noise (Figures 7A,D).

The data from the retina showed large areas with a big fraction of correlated events; these areas were the parts of the chip with good electrical coupling between the tissue and the chip (Figures 7B,E). The round area in the center was the optic disk, where the algorithm correctly detected no spikes. The areas without spikes toward the periphery either reflected incisions made to flatten the retina, or areas with poor contact. Interestingly, the density of uncorrelated spikes was higher in areas where the tissue was well coupled to the chip, suggesting the presence of further undetected events.

Finally, we found that the fraction of correlated events on an empty MEA was very low, and the noise was, as in the culture data, evenly distributed (Figures 7C,F; note that the total number of detected events was much lower in this recording; see also Figure 6D). This recording was made using the same MEA chip that was also used for the retina. A comparison of the noise levels between those recordings showed that some channels with high noise levels were comparable in both cases (arrows in Figures 7E,F). This shows that variations in recording quality between different electrodes can be expected even on a single MEA.

**Figure 8:**
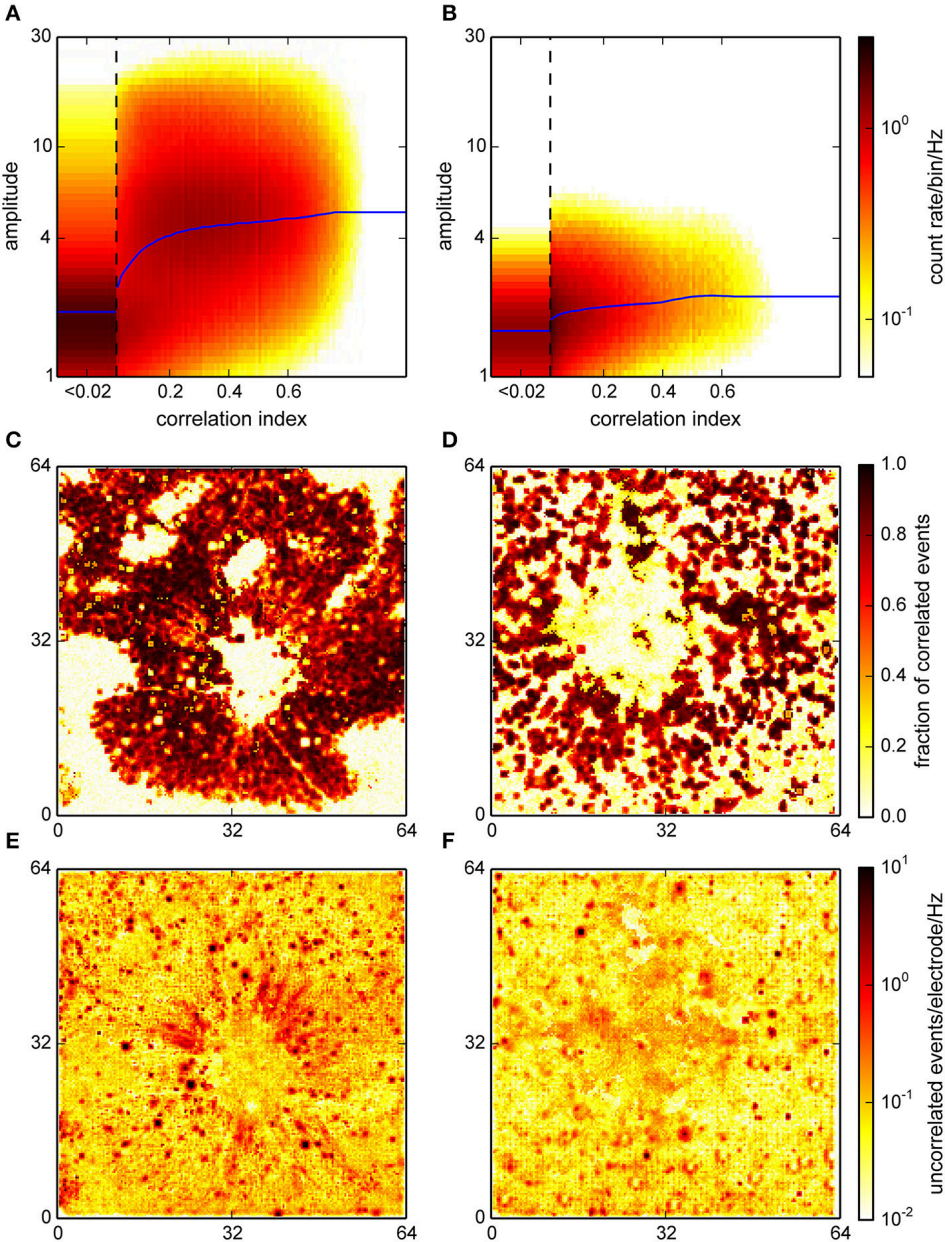
**Correlation analysis for the interpolating detection**. **(A,B)** Histograms of the joint distribution of amplitudes, expressed as a spatio-temporal average in units of the variability estimate *v* (cf. Section 2), and correlation indices for a retina **(A)** and a culture **(B)**. Blue curves represent a sliding median of event amplitudes. For the retina, this distribution was bimodal with one maximum at high correlation indices and amplitudes which likely corresponded to neural activity. **(C,D)** Spatial representation of the estimated fraction of correlated events across the active area (64 × 64 electrodes) of the chip. **(E,F)** Spatial density of uncorrelated events. This again clearly identified spots of uncorrelated activity corresponding to noisy electrodes.

We also performed the correlation analysis on the interpolated and localized spikes with a modified version of the algorithm, adapted for a higher spatial resolution and a different amplitude measure. The correlation indices assigned to each event in this procedure were qualitatively distributed as in the online detection (Figure 8B; compare with Figure 5A). Note that in particular for the retina recording (Figure 8A) where spike amplitudes were much higher than in cultures, events with higher CI were clearly clustered around higher signal amplitudes.

Moreover, correlated events were now entirely restricted to areas where activity was expected (Figures 8C,D). In the retina recording, the uncorrelated events formed radial structures near the optic disc, possibly due to axons, which were not resolved in the online analysis (Figure 8E). In the culture recording, the center region showed an increased level of uncorrelated activity, which could be indicative of the detection of signals with a poor electrical coupling (Figure 8F). This again indicated that interpolation could indeed reduce the noise that impaired detection on single channels.

To test the notion that correlated events reflected neural activity, a comparison of the anatomical image and only events that were classified as correlated (i.e., *p* > 0.5) was performed as in the previous section. We found that this restriction to correlated events removed false positives to a greater extent than a threshold increase would, but barely affected the fraction of nuclei where no adjacent cluster of spikes was found (see Figure 4B), thus confirming that correlated events reflected neural activity at least for data from spontaneously active cultures.

We conclude that correlations were present in both preparations and absent for events detected on an empty chip. Those correlations could thus help to quantify the performance of the detection for individual locations. The classification of individual events did not separate true events from noise, but could be used to characterize spike shapes or, for the low amplitude fraction, assess the presence of current sources with a weak electrical coupling. We summarized the detection and classification results for both methods in Table 2.

**Table 2:** **Events detected and classified for various preparations and methods**.

**Method**	**Threshold**		**Empty chip**		**Culture**		**Retina**	
	**O**	**I**	**O**	**I**	**O**	**I**	**O**	**I**
Detected	6	0	1.3 M/h	2.2 M/h	4.2 M/h	4.5 M/h	18.2 M/h	11.4 M/h
Isolated	6	0	1.3 M/h	2.1 M/h	3.9 M/h	4.1 M/h	8.7 M/h	10.4 M/h
Correlated	6	0	47 k/h	97 k/h	2.0 M/h	2.4 M/h	6.2 M/h	6.9 M/h
Isolated	8	2	480 k/h	500 k/h	1.6 M/h	1.6 M/h	6.5 M/h	7.3 M/h
Correlated	8	2	37 k/h	46 k/h	0.9 M/h	1.1 M/h	4.9 M/h	5.3 M/h

“*method”: online detection (“O”) or interpolating detection (“I”). “detection”: the rate of all detected events; “isolated”: remaining events after removal of doubly detected events; “correlated”: events classified as correlated. The last two rows show the effect of thresholding event amplitudes at a higher threshold*.

### 3.6. Inhomogeneities in spatial activity profiles revealed functional network properties

The results from the spatial spike localization indicated that it may be difficult if not impossible to cluster neural activity into single units for data from dense cultures. Therefore, we were interested to see whether the network activity in cultures could also be analyzed without defining single units, by directly searching for functional features in the three-dimensional spatio-temporal activity maps we obtain. In particular, here we considered relations between temporally adjacent events. In contrast to traditional approaches that attempted to analyze the activity of single neurons or MUA activity, we quantified correlations for individual spikes. This yielded two-dimensional, high resolution maps of an estimate of a directional bias to the center of mass of coincident activity.

This procedure revealed small, local structures with non-random associated network activity. These small areas likely corresponded to single or groups of neurons, and the angle indicated a local bias for the direction of the predominant network activity when spikes occurred. This often indicated whether a neuron participated in local (bias away from the center of the chip) or widespread (bias toward the center) activity. In addition, numerous local inhomogeneities in activity propagation could be seen, likely reflecting specific connectivity in the network. Cross validation using split data sets showed that these patterns were consistent within single recordings (not illustrated).

**Figure 9:**
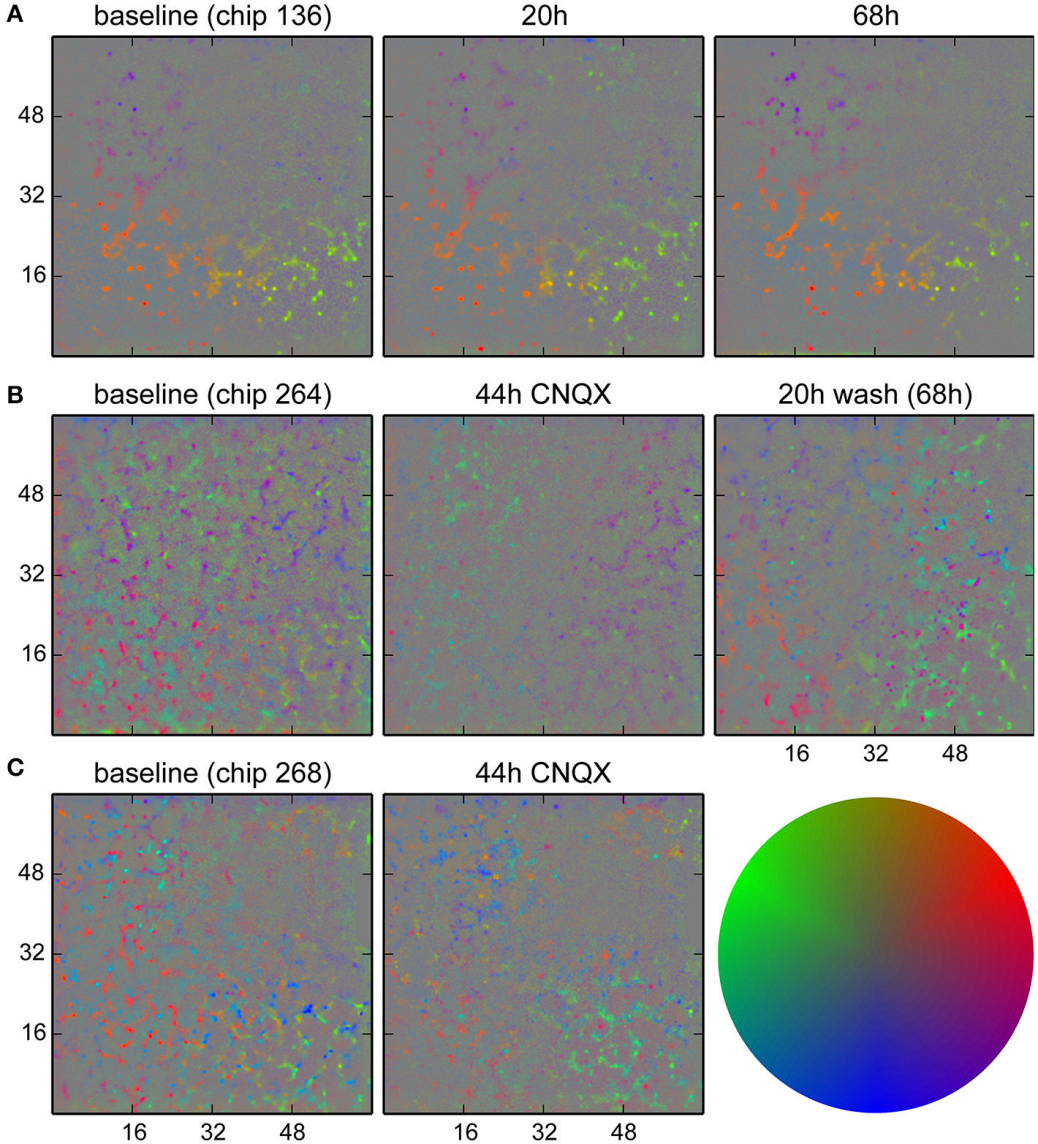
**Analysis of activity patterns in cultured networks**. Colors indicate a bias in the direction where the strongest correlated activity was found for each location. **(A)** Control culture. Some units tended to fire when the activity was less localized than activity in the surrounding. This pattern stayed for at least 68 h. **(B)** Culture where CNQX was applied and the firing pattern did change. The presence of two distinct direction biases at similar locations was likely due to units firing earlier or later during activity propagating in a wave-like fashion. During CNQX application, the firing rate was lower, and patterns were less significant. After washout of the drug, a new pattern emerged. Specifically, the purple dots on the lower right show newly active units with a tendency to fire when the network activity was highly localized. **(C)** A second culture where changes were already visible during the application of CNQX. The color code used for direction biases is depicted on the right. Gray corresponds to the absence of a bias (center of the disk) and a pure color corresponds to three standard deviations.

We monitored long term changes in network behavior for three cultures. In Figure 9A, results for a non-perturbed culture are shown, showing a set of neurons that were more active than others and whose firing happened when the population activity was less localized to that particular spot. These remained mostly identical over a 68 h period. For two cultures (Figures 9B,C), synaptic transmission was impaired by blocking AMPA receptors with CNQX (5 µM) after the baseline recording for a period of 48 h. The activity at baseline in these two cultures was more complex and showed at least two distinct groups participating in different behavior. Application of CNQX strongly reduced firing rates and disrupted the patterns in some areas. Note that for this dose of CNQX, we observed a partial recovery in network firing rates when recording after 44 h; a higher dose (10 µM) was shown not to recover (Slomowitz et al., 2015). After washout of CNQX, a new pattern of network activity was formed, where new spots of activity emerged with a tendency to participate in local activity (bias away from the center). In Figure 9C, such spots were already observed after 44 h, before washout of CNQX.

Summarizing, this analysis showed that indeed salient activity patterns could be identified and, furthermore, it was possible to distinguish single units based on their activity. Such patterns remained remarkably stable over time, but did change when the network was perturbed.

## 4. Discussion

We have presented a set of algorithms for spike detection specifically designed for extracellular recordings with high density, large scale MEAs with thousands of electrodes. The implementation of an online estimation procedure of data quantiles made these algorithms very efficient, to be able to cope with the high data rates produced by such arrays.

We adopted two strategies to detect spikes. The first method was designed to run in real-time for data recorded from 4096 channels at 8 kHz, and identified spikes based on the signals of individual channels. This approach is ideal for long term recordings (hours) where the amount of raw data is too large to be stored intermediately, and for experiments where real-time feedback is required. It can also be used to detect spikes in single or traditional MEA recordings, as it is based on a more robust noise estimate than the signal variance.

Our second method provided an estimate of the source location of each spike by exploiting signals originating from neighboring channels. This method is less sensitive to fluctuations in individual channels and thus sets a harder amplitude threshold, which enables a more reliable detection of small events from neurons not precisely co-localized with recording electrodes. The localized events can then be used to perform spike sorting based on spatial clustering, which can be supplemented by shape information to reliably separate spikes from single neurons (cf. Prentice et al., 2011; Pillow et al., 2013; Ekanadham et al., 2014). Doing a clustering at such a late stage during the processing may be less efficient than direct template matching approaches (Franke et al., 2015) but yields information about less active units and facilitates comparisons across days when spike shapes might change. This method is computationally more costly than the online method, but could potentially achieve real-time performance in a parallelized implementation.

Low-threshold detection of candidate events served to minimize the loss of true positives due to early rejection. Based on the idea that most physiological spikes would show at least a weak correlation with other events, we classified events as correlated or uncorrelated. This method is not suitable for recordings from preparations where neural firing is essentially uncorrelated, and likely underestimates the fraction of actual spikes in our recordings, where we also expect uncorrelated, isolated spikes. However, it allows for systematic comparison of the detection methods, and exposes their advantages and limitations.

This is important since a complete direct validation of our methods was not feasible because (1) we do not have the ground truth for our data and (2) we believe it is not possible to construct a realistic MEA noise model to generate synthetic data. We instead used synthetic data inserted into a recording from an empty chip and the presence of correlations for verification. We note that we also conducted this analysis with synthetic data in a retinal recording (which also contained other spikes), which exhibited essentially the same behavior as reported here for an empty chip. In this case however the additional current sources due to neural activity could not be controlled, hence the analysis of synthetic data could only be viewed as an approximation of a realistic scenario. Overall, this analysis indicated the presence of three main sources of noise:

1. Stochastic noise which was due to the thresholding and was fairly constant across all electrodes. This contribution was small in absolute terms and almost completely eliminated by a moderate increase in threshold.
2. Differences in intrinsic noise in a subset of electrodes. These were identified using an empty chip recording. The interpolating detection located events detected as a result of this noise strongly centered on the respective electrode, which was unlikely to happen for real neurons, and could thus flag them for manual inspection or cross-comparison with an empty chip recording (if available).
3. Different physiological current sources other than spikes, whose superposition was measured at the electrodes. They were modulated by network activity, and were thus not increasing the variability estimate (and hence the threshold) as much as continuous fluctuations. Specifically, neurons had their strongest current sources at the axon hillock, but this signal attenuated with distance from an electrode such that it may appear small in the recording. In addition, axons and other neural processes likely caused small, fast fluctuations, which were difficult to distinguish from true spikes. Overall, together these noise sources lead to unimodal event amplitude distributions, without clear separation of spikes and other current sources.

We also performed a comparison of the detected events with the positions of neural nuclei in cultured neurons, which revealed that both are predominantly found within 40 µm. Further away, the density of detected events did not depend on distance and therefore indicated events which were not originating from a somata or axon hillocks. Correlated events were more closely associated with nuclei and were found more frequently adjacent to nuclei than high amplitude events. Hence generally activity of some neurons was only detectable with a low detection threshold.

### 4.1. How many true spikes could we find?

Table 2 summarizes the number of events detected with both methods and the amount classified as true spikes as identified by the correlation analysis. For an interpretation of those numbers, it is important to emphasize that the analysis for the interpolating detection had a higher spatial resolution and used signals averaged over multiple electrodes.

First, we observed that a threshold increase for the interpolating detection resulted in a much higher reduction of detected events in the empty chip recording than with the neural preparations, as compared to the online detection method. This indicated that the interpolation effectively reduced stochastic noise, whereas physiological noise would be seen on more than one electrode and was therefore less affected.

Accordingly, we detected a smaller fraction of events that were classified as uncorrelated in the interpolating detection for the culture recording. As this relative decrease did not result in a reduced overall detection rate, it indicated that the interpolation improved the detection. For the retina, a higher fraction of events from the interpolating detection was classified uncorrelated, possibly due to an improved detection of events from spatially extended current sources such as axons. Further, it appeared that in both online and interpolating detection there were units which were not significantly correlated with the activity of other units, as their number greatly exceeded the number expected from the empty chip recording (compare Figure 2E and Figure 8E). This would explain why the amount of uncorrelated events remained high even after increasing the detection threshold.

In summary, this analysis suggested that the interpolation method allowed for a more consistent detection of events from specific current sources. It also showed that signals from weak sources were still detectable due to stochastic resonance, but this would have the undesired effect of yielding only an incomplete record of the activity of such neurons. This result therefore highlighted an important general caveat of performing spike detection under non-optimal signal-to-noise conditions, and showed how high density probes in combination with signal interpolation could be used to obtain more reliable and consistent spike records.

### 4.2. How to use these methods

The online method offers an efficient way to obtain estimates of the population activity, and a higher threshold can be used in order to minimize false positives. Further, in certain preparations uncorrelated, potentially noisy channels can be detected using the correlation analysis and excluded if the fraction of uncorrelated events exceeds 50%. Generally however, this method and more generally threshold-based detection on single channels does not provide sufficient information to reliably separate true spikes from noise, either true spikes are lost when the threshold is high or noise is introduced when a low threshold is used. In addition, it does not enable single unit identification in high density recordings.

In order to isolate the activity of individual units, the interpolating detection provides a new, efficient starting point. Apart from reducing a bias to detect sources centered on electrodes, it effectively reduces the noise variance at the expense of signal amplitude. Therefore, we expect units with low amplitudes to be lost and units with high amplitudes to be detected reliably with a spatial resolution higher than the density of electrodes. On the other hand, there is also an increased risk of detecting signals from spatially extended sources such as axons. These however can be identified as elongated structures and removed, and will typically show a waveform different from the typical biphasic spike events. Moreover, external influences, like a perfusion system may have an effect that has to be considered as they would induce correlated voltage fluctuations.

To validate the detection performance, the correlation analysis can be used to identify areas with a reliable detection, although this is only feasible in preparations such as cultured neurons where at least weak correlations are always present. Preliminary work indicates that clustering of such data sets, which combines location and spike shape information, can yield excellent and efficient isolation of single units in recordings from the retina (Sorbaro et al., unpublished data). In cultures, such clusters are largely overlapping, such that it appears difficult to find boundaries between clusters. However, it is possible to analyze such data without prior clustering. In this case every event needs to be compared to its surrounding activity, and a spatial map is obtained, highlighting areas with different properties.

### 4.3. Technical prospects

High density MEAs are likely to scale further with technical developments, and offer an important avenue for development of culture, slice, and explant recordings. Conventional MEAs have been used to increase the temporal resolution of calcium imaging experiments in cultures (Herzog et al., 2011; Slomowitz et al., 2015). We anticipate that high density MEAs will be more useful in such a context as they allow for a mapping between imaged and current source locations. We suggest that the algorithms presented in this paper may be readily applicable to find such a mapping. This will allow for the distinction of excitatory and inhibitory neurons, identification and characterization of sparsely firing neurons (Shoham et al., 2006) and further increase the SNR. Further, it enables continous electrical measurements with a high coverage where optical recordings need to be interrupted due to photobleaching effects.

### 4.4. Data sharing

All code to replicate the analysis shown in this paper is provided at https://github.com/martinosorb/herding-spikes, and example datasets at https://portal.carmen.org.uk/#link=URN:LSID:portal.carmen.org.uk:metadata:42944.

## Author contributions

JM, UB, and MH designed the research project. HA, AM, DP and LB performed experiments on cultures, ES performed the retina experiment. JM and MH developed software and analyzed data. JM, ES, DP, LB, UB, and MH wrote the paper.

## Funding

This work was supported by the EuroSPIN Erasmus Mundus programme and NCBS/TIFR (JM), by the European Commission for Research within the Seventh Framework Programme for the NAMASEN (FP7-264872) Marie-Curie Initial Training Network (HA), grants EP/F500385/1, and BB/F529254/1 to the University of Edinburgh Doctoral Training Centre in Neuroinformatics and Computational Neuroscience from the UK EPSRC, BBSRC, and MRC research councils (DP), the Istituto Italiano di Tecnologia (AM, LB), Biotechnology and Biological Sciences Research Council (BBSRC) grant BB/H023569/1 (ES, MH), and UK Medical Research Council Fellowship MRC G0900425 (MH).

### Conflict of interest statement

The authors declare that the research was conducted in the absence of any commercial or financial relationships that could be construed as a potential conflict of interest.
