# Supplementary material for: Spike Detection for Large Neural Populations Using High Density Multielectrode Arrays
Source: Front Neuroinform. 2015 Dec 18;9:28. doi: 10.3389/fninf.2015.00028 (PMC4683190; doi:10.3389/fninf.2015.00028)
Supplement: Supplementary file 1 [file Image1.pdf]

Supplementary Material: Spike detection for large neural populations using high density multielectrode arrays

Oliver Muthmann*, Hayder Amin, Evelyne Sernagor , Alessandro Maccione, Dagmara Panas, Luca Berdondini, Upinder S. Bhalla and Matthias H. Hennig*

*Correspondence:
Oliver Muthmann
jensoliver@ncbs.res.in

Matthias H. Hennig: m.hennig@ed.ac.uk

1 SUPPLEMENTARY TABLES AND FIGURES

The recording system that was used in this work had a inter-electrode distance d_{pitch} of 42 μm. In order to give an idea about necessary changes for different electrode distances, we illustrated dependencies in a simplified setting. To this end, we assume noise on different electrodes to be independently Gaussian distributed with equal variance. The spatial decay over a distance d (in μm) of a low-pass filtered signal amplitude was described by the following function

$$A(d) = \min\left(1, \frac{300\,(1 - \exp\left(-\max(1,d)/30\right))}{\max(1,d)^2}\right),$$

which qualitatively matches simulations in Hagen et al. (2015) and is visualized in Figure 1 A. Current sources were assumed to be distributed uniformly in space. Over a grid of 100x100 positions $x_k \quad k \in \{1, \ldots 10000\}$ of current sources in one quadrant of the area closest to an electrode (cf. Figure 1 F) , the amplitudes $A_{ik} = A(|x_k - y_i|)$ (i denotes the electrode index) for 25 electrodes (at positions y_i) were determined. The root mean squared amplitudes

$$w_i = \sqrt{\sum_k A_{ik}^2}$$

were used as weights to determine a maximum achievable weighted $\tilde{A}_k$ signal amplitude, which could be obtained by template matching. These weights were then normalized such that the amplitude of the noise of the weighted signal corresponds to the noise amplitude on a single channel, i.e.

$$\tilde{A}_k = \frac{A_{ik} w_i}{\sqrt{\sum w_i^2}}.$$

The squared weighted signal (referred to as signal power) is shown in Figure 1 D, and depends on the position x_k of the event with respect to the electrode y_i. The indices k were normalized to (0,1] to represent the fraction of the area (sorted by amplitude) with a given signal strength.

In the main paper, we adopted a strategy to predefine weights for two scenarios: one where the event is close to an electrode and one where it is in between 4 electrodes. Here, we ran an optimization to separate the space into an area that is better detected when assigning equal weights to electrodes with equal distance from the center electrode (Figure 1 B, inset) or a point centered between 4 electrodes (Figure 1 C, inset). The magnitude of the former area is shown in Figure 1 E (black line) and the spatial division in Figure 1 F, both in dependence of d_{pitch}.

Next, we compared the weighted signal power, using those two sets of weights for the respective area with the power obtained in the optimal approach where specific weights were given to each electrode (Figure 1 G,H). In Figure 1 G it can be seen that the performance is impaired at the boundary region where the two sets of weights give rise to similar amplitudes. Figure 1 H shows that the 4 and 5-channel interpolation (cyan/orange line) captures more than 80 % of the maximal power for $d_{\mathrm{pitch}} > 30\,\mu$m. For a higher electrode density, more electrodes should be used for interpolation. Additionally we show the effect on the signal when more distant electrodes are ignored. In Figure 1 I, we illustrate the resulting amplitudes when we were only using the weights from Figure 1 B for the entire area. The effect of including the 4 channel interpolation on overall signal power seems small here, but note that this increase occurs specifically in an area of lower signal power.

REFERENCES

Hagen, E., Ness, T. V., Khosrowshahi, A., Sørensen, C., Fyhn, M., Hafting, T., et al. (2015). Visapy: A python tool for biophysics-based generation of virtual spiking activity for evaluation of spike-sorting algorithms. *Journal of neuroscience methods* 245, 182–204

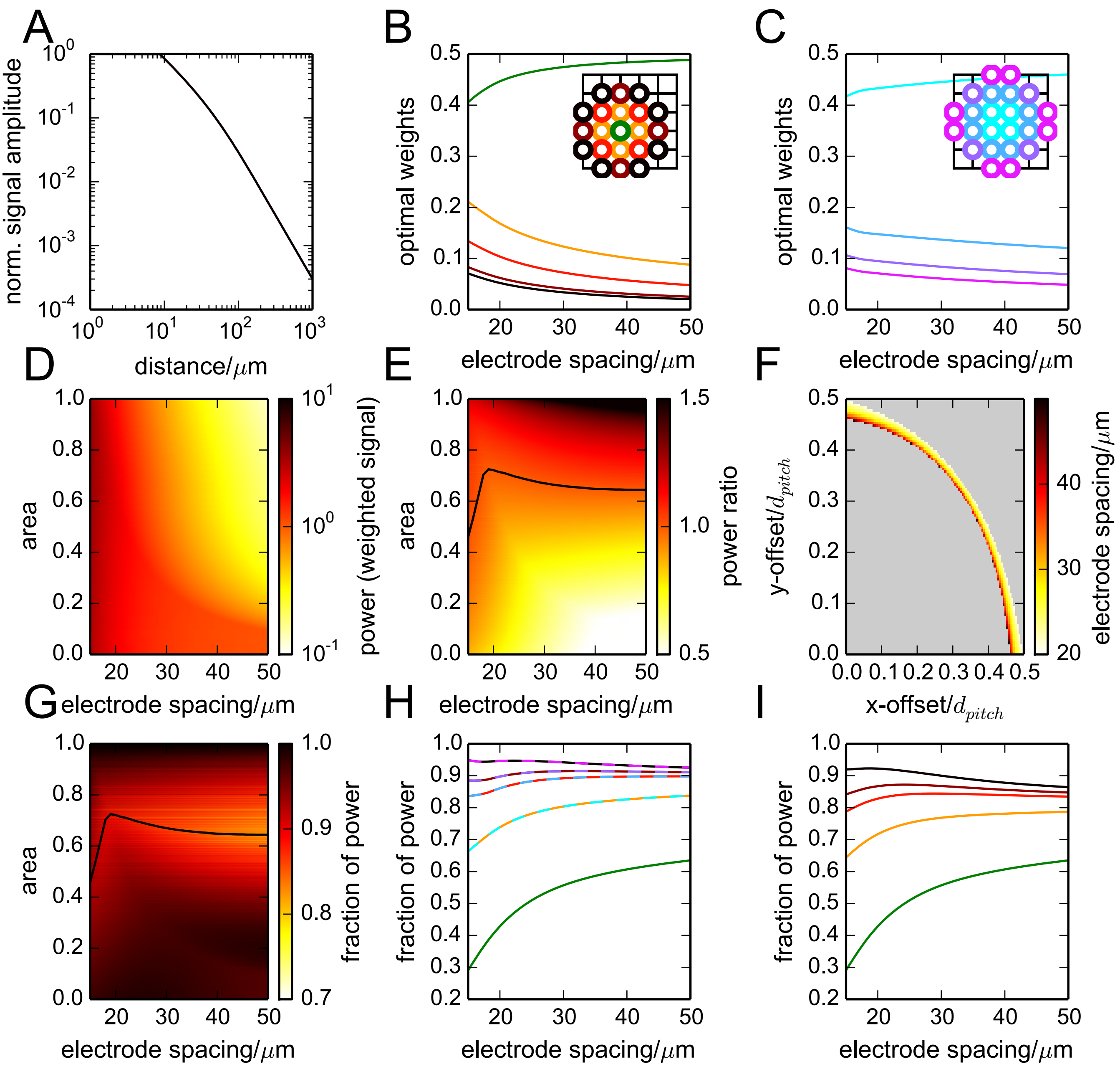

Supplementary Figure 1. Interpolation for systems with different d_{pitch}. (**A**) Assumed spatial decay of signal amplitude. (**B**) Optimal weights for detecting events close to electrodes. Electrodes with equal distance from the center electrode were given equal weights. (**C**) Same as (B) for locations between 4 electrodes. (**D**) Squared weighted signal amplitudes corresponding to a template matching approach. (**E**) Ratio of the squared signal amplitude for weights as in (C) vs. weights as in (B). (**F**) Separation line below which weights as in (B) yield higher amplitudes. (**G**) Comparison to the optimal method (D). The area is sorted as in (F), showing that overall performance is compromised where both weightings yield similar amplitudes. (**H**) Performance when using subsets of electrodes. The largest distance is shown in color, color code as in (B, C). (**I**) Performance when weights in (B) were used, i.e. events were assumed to be located on an electrode.
